# Mapping the optoelectronic property space of small aromatic molecules

**DOI:** 10.1038/s42004-020-0256-7

**Published:** 2020-02-05

**Authors:** Liam Wilbraham, Denisa Smajli, Isabelle Heath-Apostolopoulos, Martijn A. Zwijnenburg

**Affiliations:** Department of Chemistry, University College London, 20 Gordon Street, London, WC1H 0AJ UK

**Keywords:** Cheminformatics, Electronic materials, Optical materials, Computational chemistry

## Abstract

Small aromatic molecules and their quinone derivatives find use in organic transistors, solar-cells, thermoelectrics, batteries and photocatalysts. These applications exploit the optoelectronic properties of these molecules and the ease by which such properties can be tuned by the introduction of heteroatoms and/or the addition of functional groups. We perform a high-throughput virtual screening using the xTB family of density functional tight-binding methods to map the optoelectronic property space of ~250,000 molecules. The large volume of data generated allows for a broad understanding of how the presence of heteroatoms and functional groups affect the ionisation potential, electron affinity and optical gap values of these molecular semiconductors, and how the structural features – on their own or in combination with one another – allow access to particular regions of the optoelectronic property space. Finally, we identify the apparent boundaries of the optoelectronic property space for these molecules: regions of property space that appear off limits for any small aromatic molecule.

## Abstract (web summary)

Computational methods are a powerful tool for rapidly searching the optoelectronic property space of organic molecules. Here the property landscape of around 250,000 arenes and quinones is systematically explored, providing guidance for the design of new functional organic molecules.

## Introduction

Small aromatic molecules and their quinone-derivatives find application in the solid-state as molecular semiconductors, forming the semiconducting channel of organic field effect transistors^1–4^, the light absorbing layers of organic solar cells^5,6^, and organic thermoelectric materials^7,8^ for thermoelectronic generators, molecular dopant for such devices^1,3^, as well as dyes for dye-sensitised solar-cells^9^ and luminescent spectral converters/concentrators^10^. The same types of molecules, when in solution, find use as fluorescence sensors^11,12^, photoredox catalysts for organic synthesis^13^, and as redox flow battery analytes and/or catholytes^14^. All of these applications exploit the (opto)electronic properties of these molecules and the ease by which these properties can be tuned by the introduction of heteroatoms, e.g., replacing a –CH– group in a benzene ring by a nitrogen atom, or by the addition of functional groups, e.g., replacing hydrogen atoms by electron donating amino (-NH_2_) or electron withdrawing nitro (-NO_2_) functional groups. This tuning, in principle, allows for the simple navigation of relevant property spaces, but also presents challenges when aiming to understand trends, cooperative effects, and the limits to the kind of properties that can be achieved for small molecules. In turn, this makes the task of finding “optimal” molecules for specific applications more difficult. When increasing the number of unsubstituted molecular skeletons, possible heteroatoms, and/or functional groups in a screen, the amount of molecules to consider explodes quickly into the hundreds of thousands or even millions. Hence, it is near impossible to explore a significant part of the property space of such small aromatic molecules experimentally, even when using robotic synthesis and characterisation platforms or using existing literature data. Also, using standard computational techniques, such as density functional theory (DFT), the large number of molecules makes it computationally costly to properly sample their property space, even if calculations on individual molecules are still relatively inexpensive, and to provide context to trends in a data-driven manner.

**Fig. 1:**
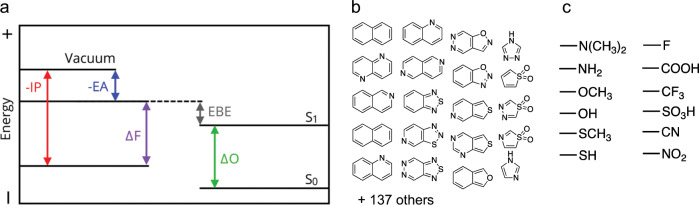
Optoelectronic properties and data set. **a** Illustration of the relationships between the negative of the ionisation potential (−IP) and electron affinity (−EA), fundamental gap (ΔF), exciton binding energy (EBE), and optical gap (ΔO); **b** a selection of the molecular skeletons used in the study; **c** the substituents used in the study.

Here we use the computationally efficient density functional tight-binding method—GFN-xTB—developed by Grimme and co-workers^15^, and its IPEA-xTB^16^ and sTDA-xTB^17^ extensions, to calculate (see Fig. 1a) the negative of the ionisation potential (-IP), and electron affinity (-EA), as well as the optical gap (ΔO), of a set of ~250,000 small molecules formed by combining 157 aromatic or non-aromatic quinone molecular skeletons with up to 30 non-hydrogen atoms (see Fig. 1 and Supplementary Fig. 1) and up to 2 of 12 possible substituents (see Fig. 1c). By design, we avoid aromatic substituents and molecular skeletons that contain two or more aromatic sub-skeletons linked only by a single (carbon–carbon) bond. Such oligomers, oligomer-like molecules and polymers have been studied previously elsewhere^18–27^. The library of skeletons used was chosen to systematically include as many heteroatom replacement patterns as possible within the skeletons, while a specifically written *Python* script using *RDKit* http://www.rdkit.org functionality generated all possible substituent isomers and removed all symmetry equivalent duplicates. The diversity of the data set, in terms of the different skeletons and substituents, distinguishes this work from previous high-throughput virtual screening studies of small aromatic and/or quinone molecules, which focussed on narrower sub-classes of molecules for particular applications, e.g., tetra-azapentacenes as electron acceptors for organic solar cells^28^ and (hydro)quinones for redox flow batteries^29^. At the same time our exclusive focus on aromatic molecules and a number of quinone derivatives, as well as the size of the molecules, differentiates the data set used here from those developed for the training of machine learning models by Von Lilienfeld and co-workers^30,31^. (IPEA/sTDA-)xTB has a reduced computational cost relative to DFT of at least three orders of magnitude while at the same time providing a similar accuracy^32^, especially after calibrating the xTB data to DFT data for a sub-set of structures. We limit our description of condensed-phase effects to those due to dielectric screening, present in both solution and the solid-state, via the use of an implicit solvation model. In the case of the solid-state, packing effects will further perturb the calculated -IP, -EA, and Δ*O* values for the molecules. However, even in the presence of such packing effects the trends predicted here should generally hold true for solid-state materials.

Using the large data-set at our disposal we analyse the effect of substituents and heteroatoms on the −IP, −EA, and Δ*O* values of small aromatic and non-aromatic quinone molecules. We discuss the distributions of these properties, including the maxima and minima values they take. We also discuss how the substituents interact with the inherent optoelectronic properties of the molecular skeleton they decorate and how the presence of heteroatoms influences −IP, −EA, and Δ*O* values to different degrees. We demonstrate how the synergetic combination of substituents and heteroatoms gives access to a region of property space not accessible by either on their own. Finally, we examine the most prevalent molecular skeletons in different regions of the property space and discuss our results in the context of the (possible) applications of these molecules.

## Results

### *xTB* calibration

**Fig. 2:**
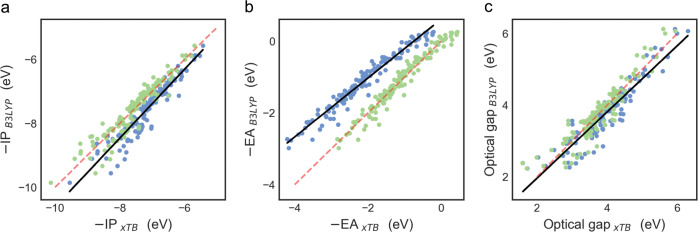
xTB calibration. Correlation between **a** −IP, **b** −EA, and **c** optical gap values as calculated with (IPEA/sTDA-)xTB and (TD-)B3LYP/aug-cc-pVTZ for the molecular skeletons. In every panel the black line is the line of best fit used to calibrate the (IPEA/sTDA-)xTB to the (TD-)B3LYP data, the red dashed line is the *x* = *y* line, the blue points are the uncalibrated data and the green points their calibrated counterparts.

Figure 2 show the correlation between the −IP, −EA, and optical gap values for the unsubstituted skeletons as calculated by (IPEA/sTDA-)xTB and those predicted by (TD-)DFT (B3LYP/aug-cc-pVTZ). Similar plots using B3LYP/DZP and ωB97x/aug-cc-pVTZ instead of B3LYP/aug-cc-pVTZ can be found as Supplementary Figs. 2and 3, while all raw data is collated in Supplementary Data 1. As previously observed by us for polymers^32^, there is a good correlation between the xTB and (TD-)DFT results, although there is a clear rigid shift between both data-sets, especially for −EA. Also a larger basis-set is required for the DFT calculations for these small molecules compared to the polymers. Using the (TD-)B3LYP/aug-cc-pVTZ data we fitted a linear model that calibrates the xTB predictions to those predicted by DFT (mean absolute error of the calibrated xTB results relative to their DFT counterparts −IP 0.20 eV, −EA 0.12 eV and Δ*O* 0.21 eV, see Supplementary Table 1 for the parameters of the linear model). All xTB results discussed in the remainder of this paper are calibrated xTB results.

### Optoelectronic property space of small molecules

**Fig. 3:**
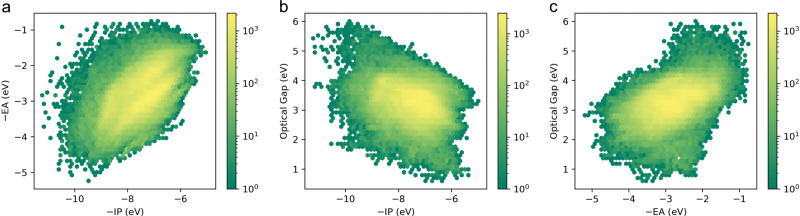
Optoelectronic property space of small molecules. 2D histograms of the property spaces spanned by **a** −IP and −EA, **b** −IP and optical gap, **c** −EA and optical gap.

Figure 3 shows projections of the optoelectronic property space of the small organic molecules, the 3D vector space spanned by the molecules’ −IP, −EA, and Δ*O* values, projected on 2D surfaces spanned by (i) −IP and −EA, (ii) −IP and Δ*O* and (iii) −EA and Δ*O*. Comparing these projections to those predicted for conjugated homopolymers and binary co-polymers^25^, a striking observation is that both property spaces appear relatively similar, suggesting that small aromatic molecules can reproduce most combinations of −IP, −EA, and Δ*O* values of conjugated polymers. This is perhaps especially surprising for the low Δ*O* region, which one naively could have supposed required extended conjugation. Another striking observation is the wide range in −IP and −EA values that molecules are predicted to display. Figure 3 also demonstrates, that while all properties are correlated, there is at least reasonable scope to independently tune any pair of −IP, −EA, or ΔO.

### Effect of substituents

**Fig. 4:**
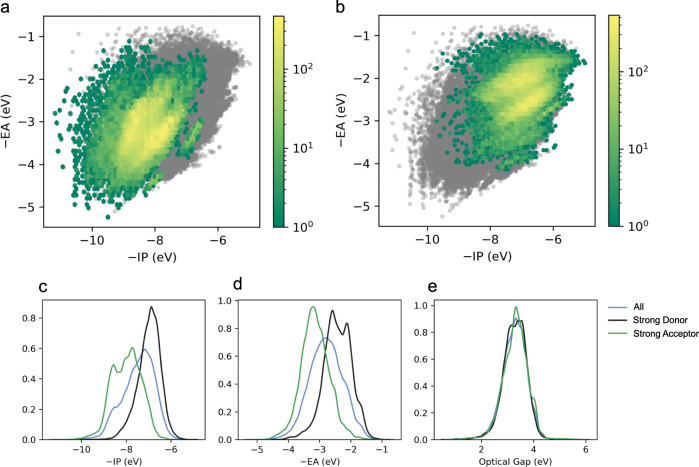
Effect of substituents. 2D histograms of the property spaces spanned by −IP and −EA in the case of strong acceptors (**a**) and strong donors (**b**); corresponding kernel density estimate (KDE) plots (**c**, **d**), as well as that for the optical gap (**e**).

Figure 4a, b shows the −IP/−EA projections of the property landscape for molecules functionalised with strongly electron withdrawing (acceptor, −NO_2_, −CN, −S(O_2_)OH, −CF_3_) and electron donating (donor, −NH_2_, −N(CH_3_)_2_, −OH, −OCH_3_) groups, respectively (see Supplementary Figs. 4–27 for plots of the individual substituents). As expected the centre of mass of the distribution for molecules with strongly electron withdrawing substituents lies in the bottom left, deep −IP/deep −EA, corner and that of molecules with strongly electron donating substituents in the top right, shallow −IP/shallow −EA, corner, and both distributions naturally divide the property space in two regions. This is made even clearer by kernel density estimate (KDE) plots of the distributions of the −IP and −EA for strongly electron withdrawing and donating substituents in Fig. 4c, d. In contrast, as can be seen from Fig. 4e the substituents have no clear systematic effect on the distribution of ΔO values.

**Fig. 5:**
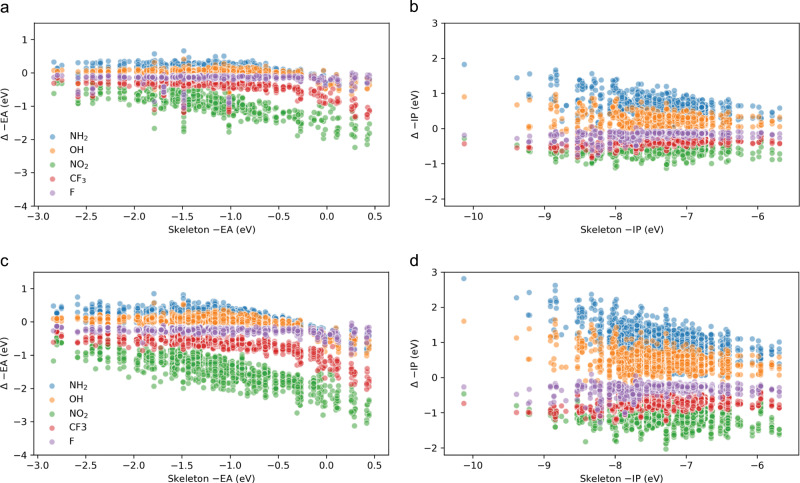
Effect of the inherent properties of the molecular skeletons. Change in −EA (**a**) and (**c**) and −IP (**b**) and (**d**) relative to that of the unsubstituted molecular skeleton after the addition of one (**a**) and (**b**) or two (**c**) and (**d**) substituents.

Figure 5 shows the change in −IP and −EA when adding one or two selected strongly electron withdrawing (−NO_2_, −CF_3_) and strongly electron donating (−NH_2_, −OH) substituents to a skeleton. Clearly the effect of adding substituents is not constant and depends on the inherent electronic properties of the skeleton. The effect of adding substituents on −IP is largest for the skeletons with the inherently deepest −IP values, while the effect on −EA is the largest for the skeletons with the inherently shallowest −EA values. Moreover, the shift in −IP is the largest for electron donating substituents and in the case of −EA for electron withdrawing substituents. The effect of substituents is hence the largest when it counteracts the natural electron richness or poverty of the skeletons. Comparing the plots for molecules with one and two of the same substituents it is clear that adding more electron withdrawing or electron donating substituents increases the size of the change in −IP and −EA but that the effect is less than additive.

### Effect of heteroatoms

**Fig. 6:**
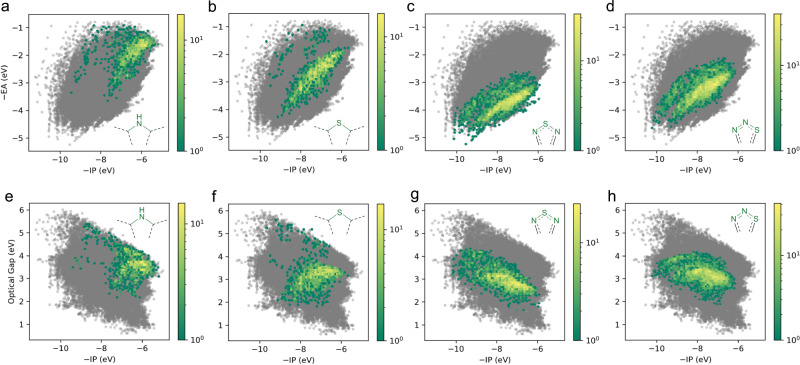
Optoelectronic property space for different heteroatom fragments. 2D histograms of the property spaces spanned by −IP and −EA for the **c[nH]c** (**a**), **csc** (**b**), **nsn** (**c**), and **nns** fragments (**d**); 2D histograms of the property spaces spanned by −IP and optical gap for the **c[nH]c** (**e**), **csc** (**f**), **nsn** (**g**), and **h** **nns** fragments.

Next, by calculating Morgan extended connectivity fingerprints^33^ for all molecules in our data set using *RDKit*, we analysed the effect of the presence of heteroatoms on the optoelectronic properties. We searched our data set for the presence of a number of heteroatom-centred SMILES fragments with three non-hydrogen atoms in the ring system (e.g., **c[nH]c**, **c[nH][cH**], or **[cH][nH][cH]** for a pyrrolic nitrogen, see the supplementary method section for a discussion of the SMILES notation used), and then mapped the property space spanned by molecules containing each of these fragments. The resulting plots can be seen in Fig. 6 and Supplementary Figs. 28–85. Of the fragments considered, molecules containing pyrrolic nitrogen are predicted to have the shallowest −EA values, followed by molecules containing thiophenic sulfur. In contrast molecules containing the thiodiazole (**nsn** or **nns**), oxodiazole (**non**) and bisimide (**cn(C)c**) fragments are predicted to have the deepest −EA values, which is in line with molecules based on these fragments finding use as non-fullerene electron acceptors for organic solar-cells^5,6^. The effect of heteroatoms on the distribution of −IP values in contrast appears less strong. In the case of Δ*O*, molecules containing quinone groups (e.g., **CC(=O)C**), such as derivatives of benzoquinone (molecule 243,647, see Supplementary Data 2) and 1,4-naphtoquinone (molecule 243894, see Supplementary Data 2), are predicted to lie in the low Δ*O* region. Another prominent skeleton in this region of property space is azulene (molecule 241,480, see Supplementary Data 2). This, however, we do not pick up directly from the fingerprint analysis due to the absence of heteroatoms in the azulene skeleton. Not surprisingly, both quinones and azulene-derivatives form the basis of dyes and pigments, including selected examples of dyes used in dye-sensitised solar-cells^34–36^ and as light absorber in organic solar-cells^37,38^. Overall it appears that incorporation of heteroatoms has a less predictable or systematic effect than adding electron withdrawing or donating substituents.

We can also pick up on the effect of isomerism in the heteroatom fragment in our data set. For example, Fig. 6c, d, g, h show the property spaces of molecules containing **nsn** and **nns** fragments, corresponding to two isomers of the thiodiazole ring. It is apparent that the molecules containing the symmetric **nsn** fragment on average have deeper −EA values than those containing the asymmetric **nns** fragment but otherwise are very similar in terms of properties.

**Fig. 7:**
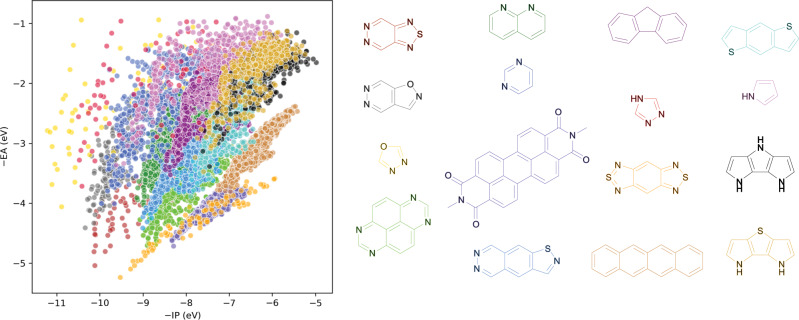
Topographical analysis of molecular skeletons. The −IP vs. −EA property space is divided into equal sub-spaces defined by increments of −IP and −EA (Supplementary Table 2). For each sub-space, we identify the most prevalent molecular skeleton—normalised by its frequency in the total dataset—and plot all substituted molecules containing that skeleton. The prevalent skeletons are shown in colour and the −IP/−EA values of the corresponding substituted molecules are shown in the same colour.

We also performed a topographic analysis, where we found the most prevalent skeleton in different regions of the property spaces spanned by −IP and −EA (Fig. 7). As expected from the discussion above, for the top right shallow −IP and shallow −EA corner of the property space the most prevalent skeletons contain pyrrolic nitrogens and those in the bottom left deep −IP and deep −EA corner thiodiazole and oxodiazole rings. In contrast, in the middle of the property space lie skeletons that contain one or more pyridinic nitrogens. These pyridinic nitrogens are often associated with deep −IP values but occur in skeletons for most of the −IP range. The latter appears to tie in with the observation above that the effect of heteroatoms on the distribution of −IP values appears less strong than for −EA.

### Effect of the number of aromatic rings

**Fig. 8:**
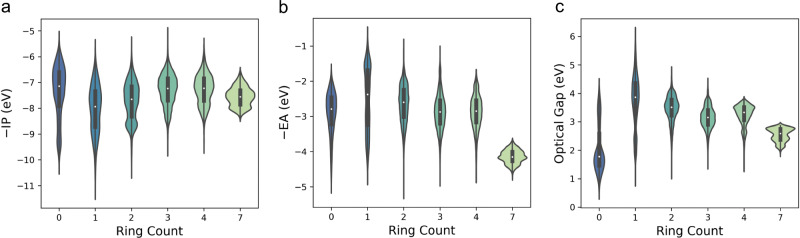
Variation in −IP, −EA and optical gap with the number of aromatic rings. Plots of −IP (**a**), −EA (**b**), and optical gap (**c**) as a function of the number of aromatic rings in a molecule.

We also investigated the effect of the number of aromatic rings in a molecule on its properties. Figure 8 show plots of the distributions of ΔO, −IP and −EA as a function of the number of aromatic rings as calculated using *RDKit*. Ignoring, in the first instance, the distribution for the molecules with zero aromatic rings, the expected trends are observed, where ΔO decreases, −IP becomes less negative and −EA more negative with increasing number of aromatic rings. The case of the molecules with zero aromatic rings corresponds to azulene and its derivatives, where the overall molecule is aromatic but the two constituting rings are not, and non-aromatic benzoquinone and its derivatives.

## Discussion

**Fig. 9:**
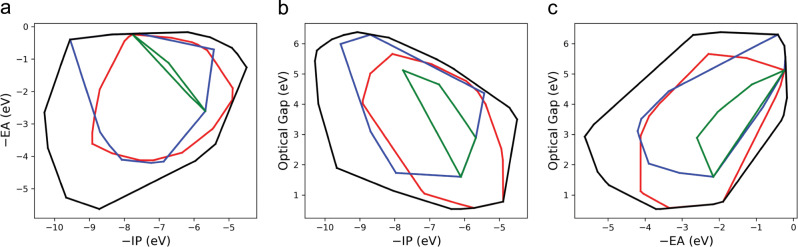
Comparing subsets of molecules. Convex hulls of the property spaces spanned by **a** −IP and −EA, **b** −IP and optical gap, **c** −EA and optical gap for the hydrocarbon molecular skeletons (green), all molecular skeletons (blue), functionalised hydrocarbon molecular skeletons (red) and all molecules in our data set (black).

We can consider the global topography of the property space of small aromatic molecules by considering the convex hulls shown in Fig. 9, which enclose the property space occupied by certain sub-sets of molecules. The first thing apparent from these convex hulls is the relatively small region of property space predicted to be occupied by the hydrocarbon molecular skeletons (green convex hull). Including molecular skeletons that contain heteroatoms (blue convex hull) or substituting some of the hydrogen atoms of the hydrocarbon skeletons with functional groups (red convex hull), is predicted to dramatically enlarge the fraction of property space covered. Both sub-sets are comparable in terms of the −EA range they allow access to but switching from pure hydrocarbon to heteroatom-containing skeletons gives access to deeper −IP values, while adding functional groups allows for molecules with more shallow −IP values. Similarly, substituting hydrogen atoms by functional groups is predicted to allow access to lower optical gap values than possible with unsubstituted skeletons, while skeletons containing heteroatoms give access to higher optical gap values. The former is not in contradiction with the observation discussed above that the substituents have no clear systematic effect on the distribution of optical gap values as the optical gap lowering effect seems to be limited to amino or alkylamino groups and most likely finds it origin in the lowest excitation for the substituted systems having *n* → π* rather than π → π* character. Comparing the red and blue convex hulls with their black counterpart, the convex hull that encloses all molecules studied, demonstrates that combining molecular skeletons with heteroatoms and substituents significantly further increases the fraction of property space covered. This includes an −EA range that appears fundamentally inaccessible by molecules belonging to the subsets of molecules discussed above. The comparison also demonstrates that the effect of including heteroatoms in the molecular skeletons and replacing one or more hydrogen atoms by functional groups can be strongly synergetic.

Good examples of the synergetic effect of substituents and heteroatoms are derivatives of benzobis(thiadiazole) and thiadiazolopyridazine with strongly electron withdrawing substituents such as nitro and cyano groups, which are the molecules with the deepest −EA values in our data set. These molecules are predicted to have much deeper −EA values than the corresponding unfunctionalised molecular skeletons (molecules 235,795 and 152,987, respectively, see Supplementary Data 2) and dinitro or dicyano derivatives of aromatic hydrocarbon skeletons (e.g., 1,4-dinitrobenzene, molecule 31, see Supplementary Data 2). For molecules with the deepest −IP values in our data set, all derivatives of the 1,3,4-oxadiazole skeleton (molecule 3283, see Supplementary Data 2) functionalised with strongly electron withdrawing substituents, the predicted difference in −IP value with the skeleton is much smaller. The same holds true for the molecules with the shallowest −IP values in the data set, derivatives of 4,7-dihydro-1H-dipyrrolo[3,2-b:2′, 3′-d]pyrrole (molecule 49,226, see Supplementary Data 2) with strongly electron donating substituents. The subset of molecules with the shallowest −EA values, finally, is predicted to contain unfunctionalised skeletons (e.g., furan, molecule 2345, see Supplementary Data 2) and even hydrocarbon skeletons (e.g., benzene, molecule 0, see Supplementary Data 2). Ultimately, even when combining the effect of heteroatoms and substituents some regions of property space remain off limits. There are no molecules that combine a shallow −IP, > −6 eV, and a deep −EA, < −4 eV, a very shallow −EA, > −1 eV, and a low optical gap, <2 eV, or a deep −EA, < −4 eV, and a large optical gap, >4 eV. The number of molecules considered in this study suggests that these might be fundamental limits to what is achievable with small aromatic or quinone molecules rather than oligomers or polymers. Switching to even larger aromatic skeletons than those studied here might offer a way to go beyond these limits but such skeletons will be synthetically challenging and may lack electronic stability^39–44^.

We can analyse what our results mean in terms of the applications molecules could be used for. Clearly quinones and azulene-derivatives, the molecules with the smallest Δ*O* values in the data-set, absorb light in the visible and thus can find use as dyes or the light-absorbing layer for organic solar-cells. Something they, as discussed above, are indeed already used for at the moment^34–38^. Amino and alkylamino substituent additionally can be used to reduce Δ*O* and shift the absorption spectrum into the red for such applications by switching the lowest excitation from π → π^*^ to *n* → π^*^. Applications that depend on molecules accepting additional electrons require deep −EA values. As discussed above, the deepest −EA values are achieved for molecules based on thiadiazole and oxodiazole rings, which are picked up in the topographic analysis in Fig. 7 as the most prevalent skeletons in the deep −EA region, in combination with electron withdrawing substituents. Such molecules could be possible alternatives to tetracyanoquinodimethane derivatives as p-dopants^1,3^ for organic transistors and thermoelectrics. They also could be used as non-fullerene electron acceptors for organic solar-cells and in fact are, as discussed above, already use for this purpose in some cases^5,6^. Applications that depend on molecules donating rather than accepting additional electrons require shallow −IP values, the shallowest of which in our data-set are achieved for skeletons containing pyrrolic nitrogen. Such molecules are unlikely to be useful as n-dopants^1,3^ for most polymers as those would have even shallower −EA values. Indeed, organic n-dopants are generally open-shell radicals^1,45^ rather than the closed-shell molecules studied here. However, shallow −IP molecules containing pyrrolic nitrogen atoms should be able to form charge-transfer salts^46^ in combination with the deep −EA molecules containing thiadiazole and oxodiazole rings. Finally outside the classical molecular semiconductor sphere, applications that depend on the molecule to reduce or oxidise other molecules after reduction, oxidation or excitation, require deep −IP values in the case of oxidation and shallow −EA values in the case of reduction chemistry. For example, molecules based on 4,7-dihydro-1H-dipyrrolo[3,2-b:2′,3′-d]pyrrole (molecule 49,226, see Supplementary Data 2), which combine relative shallow −EA values with relatively small ΔF (difference between −IP and −EA) and Δ*O* values might make good reducing photoredox catalysts^13^. This would be especially true if the substituents switch the lowest excitation from π → π^*^ to n → π^*^, reducing the adiabatic excitation energy.

In summary, we have demonstrated how the very large amount of data generated using high-throughput virtual screening allows for a data-driven mapping between structural features of small aromatic molecules and their optoelectronic properties. This mapping can be used to rationalise the optoelectronic properties of these molecules, their limits, and guide the design of molecules for applications. Specifically, we show that access to a significant region of property space of small aromatic and quinone molecules is only unlocked by combining the addition of substituents and the incorporation of heteroatoms. We also demonstrate that the effect of adding substituents depends strongly on the inherent electronic properties of the skeleton, that heteroatoms have a stronger effect on −EA than −IP and that the effect of substituents is more systematic and predictable. Finally, we discuss the most prevalent skeletons in different regions of the property space of small aromatic and quinone molecules.

## Methods

### Starting structures

The starting structures for the molecules were prepared by functionalising the SMILES representation of the molecular skeletons with up to two of twelve functional groups using the smilescombine *Python* script, https://github.com/zwijnenburggroup/smilescombine which takes functionality from *RDKit* (http://www.rdkit.org). *RDKit* was then subsequently used to embed the functionalised SMILES in three-dimensions using the EKTDG^47^ conformer generator and produce the input *xyz* coordinates for the subsequent xTB calculations. In each case 30 conformers were generated, and the energy of the conformer ranked using the MMFF94 force field^48,49^, where the lowest energy conformer is passed to xTB.

### xTB calculations

All xTB^15^ and IPEA-xTB^16^ calculations were performed using version 5.6.4SE of the *xTB* code (https://github.com/grimme-lab/xtb) and used the benzene GBSA solvation model to approximate the dielectric environment of the molecules in both the solid-state and non-polar solvents. The sTDA^17^ calculations used to predict the optical gap of the molecules were performed with version 1.5 of the *sTDA* code (https://github.com/grimme-lab/stda). These sTDA calculations take the xTB/GBSA(benzene) orbitals as input but ignore any solvation contribution when calculating ground-excited state couplings.

### (TD-)DFT calculations

All (TD-)DFT calculations used the B3LYP^50–53^ density functional in combination with the DZP^54^ or aug-cc-pVTZ^55,56^ basis-sets and the COSMO^57^ solvation model (*ε*_r_ 2.0) and were performed using Turbomole 7.01.

### Fingerprinting

We group together structures containing particular heteroatom environments by fingerprinting all the molecules in our database using the Morgan Extended-connectivity fingerprints^33^ as implemented in *RDKit* http://www.rdkit.org. with a radius of 1 and a bit-length of 4096. This procedure results in 413 unique fragments, of which after removing all the radius 0 fragments and by focussing only on fragments with three non-hydrogen atoms in the ring system and a heteroatom as the central atom or a carbon atom connected to a heteroatom that is only connected to that carbon (e.g., as in –C(=O)–) as a central atom, only 29 fragments remain. In the supplementary method section the SMILES notation of the heteroatom fragments is discussed in more detail.

### Most prevalent molecular skeletons

To identify molecular skeletons that can be used to target specific areas of the property landscape, we perform a “topographical” analysis using the unsubstituted molecular skeletons. To do so, the −IP vs. −EA property space is divided into equal sub-spaces defined by increments of −IP and −EA. For each sub-space, we identify the most prevalent molecular skeleton—normalised by its frequency in the total dataset—and plot all substituted molecules containing that skeleton.

## Supplementary information

Supplementary Information

Description of Additional Supplementary Files

Supplementary Data 1

Supplementary Data 2

## Data Availability

The authors can confirm that the data supporting the findings of this paper are available within the paper and its supplementary information (Supplementary Data 1 and 2).

## Code availability

The code for generation of the substituted SMILES can be found at https://github.com/zwijnenburggroup/smilescombine. The IPython notebook used for the analysis of the data and preparation of the figures in the main text and supporting information can be found at: https://github.com/zwijnenburggroup/2019-small-molecule-mapping.
