# Supplementary material for: Mapping the optoelectronic property space of small aromatic molecules
Source: Commun Chem. 2020 Feb 5;3:14. doi: 10.1038/s42004-020-0256-7 (PMC9814262; doi:10.1038/s42004-020-0256-7)
Supplement: Supplementary file 1 — Supplementary Information [file 42004_2020_256_MOESM1_ESM.pdf]

Supplementary information for

Mapping the optoelectronic property space of small aromatic molecules

Liam Wilbraham,[1] Denisa Smalji,[1] Isabelle Heath- Apostolopoulos,[1] Martijn A. Zwijnenburg[1,*]

Department of Chemistry, University College London, 20 Gordon Street, London WC1H 0AJ, UK

[*] E-mail: m.zwijnenburg@ucl.ac.uk

Supplementary figures

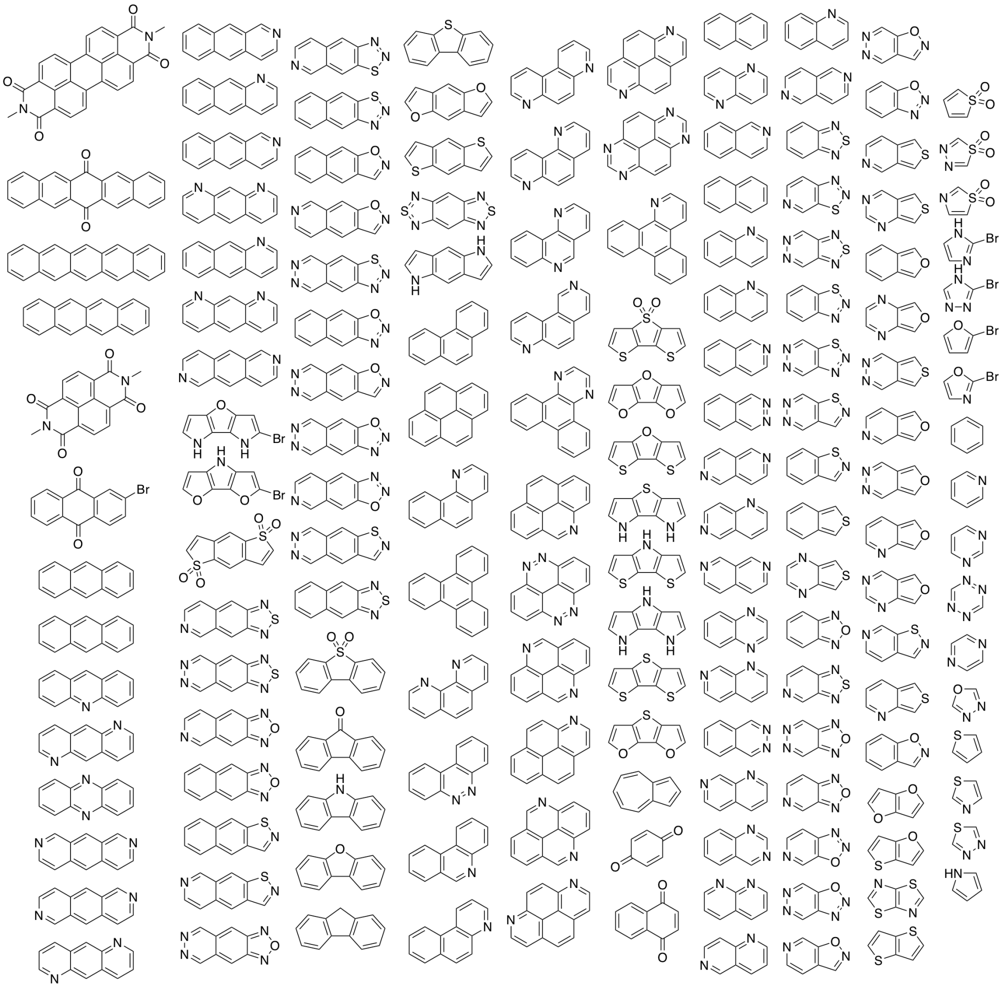

Supplementary Fig. 1 All the molecular skeletons included in the study.

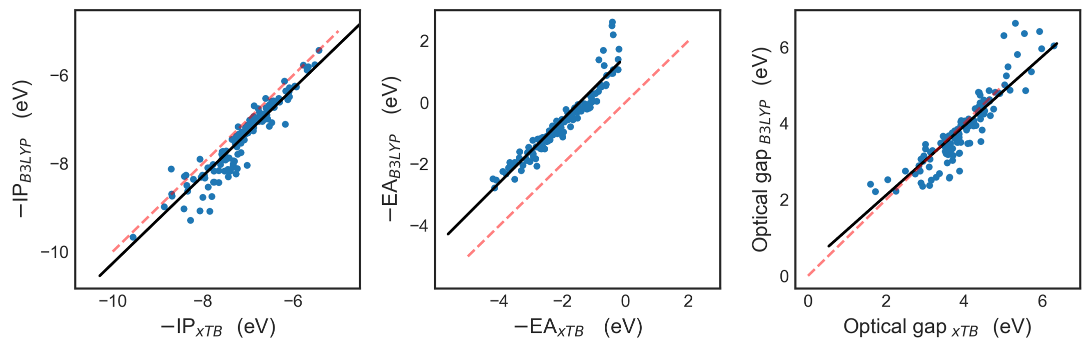

Supplementary Fig. 2 Correlation between –IP (left), –EA (centre) and optical gap values (right) as calculated with (IPEA/sTDA-)xTB and (TD-)B3LYP/DZP for the molecular skeletons. In every panel the black line is the line of best fit used to calibrate the (IPEA/sTDA-)xTB to the (TD-)B3LYP data while the red dashed line is the x = y line.

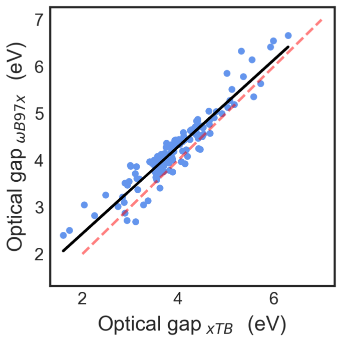

Supplementary Fig. 3 Comparison of the Δ_0 values calculated for the molecular skeletons using sTDA-xTB and ωB97x/aug-cc-pVTZ.

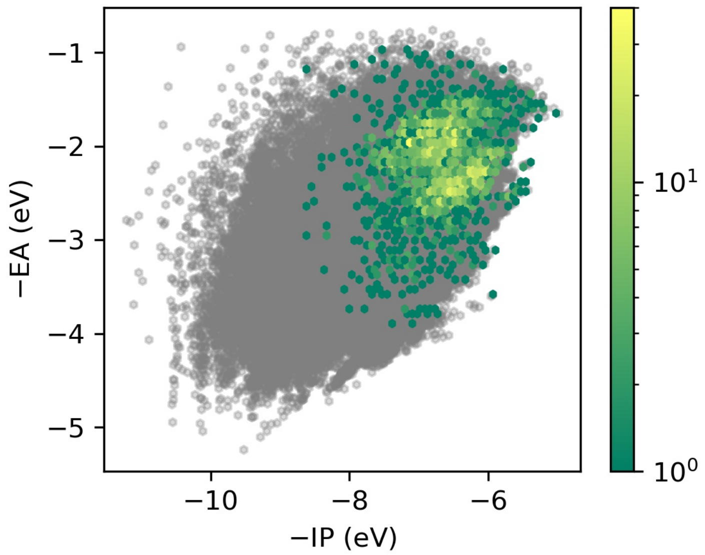

Supplementary Fig. 4 2D histogram of the property space spanned by -IP and -EA for molecules functionalised with one or two NH_2 groups.

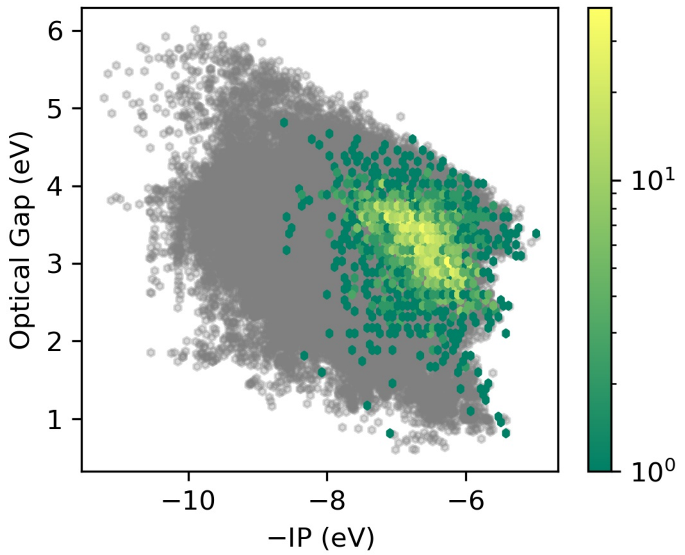

Supplementary Fig. 5 2D histogram of the property space spanned by -IP and the optical gap for molecules functionalised with one or two NH_2 groups.

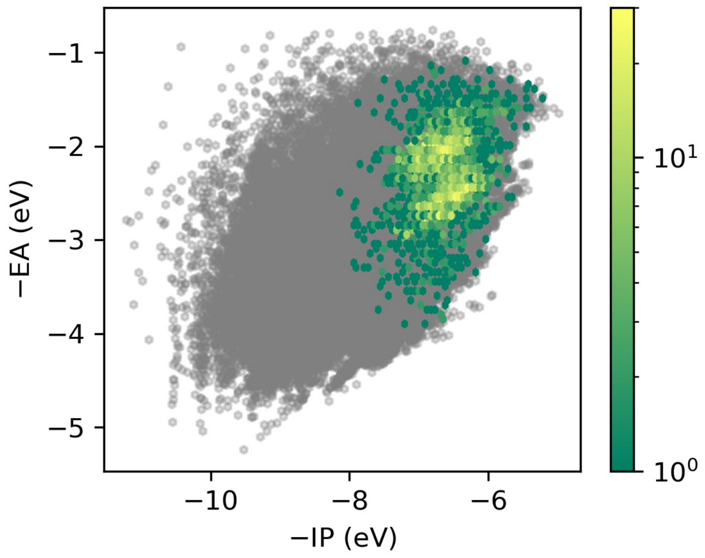

Supplementary Fig. 6 2D histogram of the property space spanned by -IP and -EA for molecules functionalised with one or two $N(CH_3)_2$ groups.

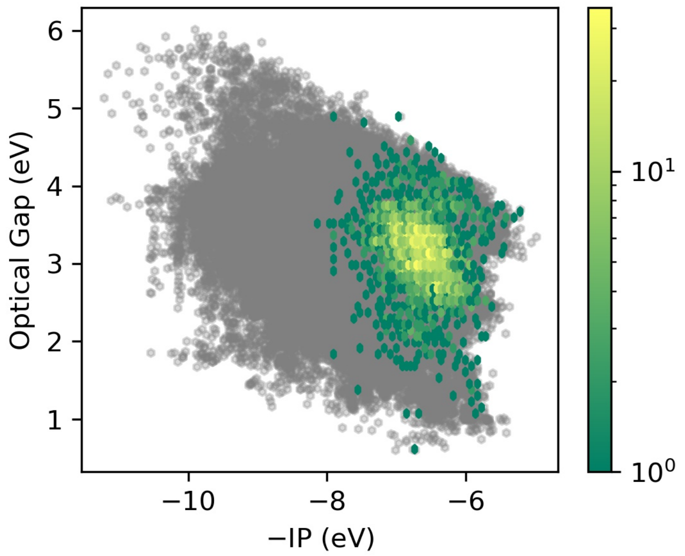

Supplementary Fig. 7 2D histogram of the property space spanned by -IP and the optical gap for molecules functionalised with one or two $N(CH_3)_2$ groups.

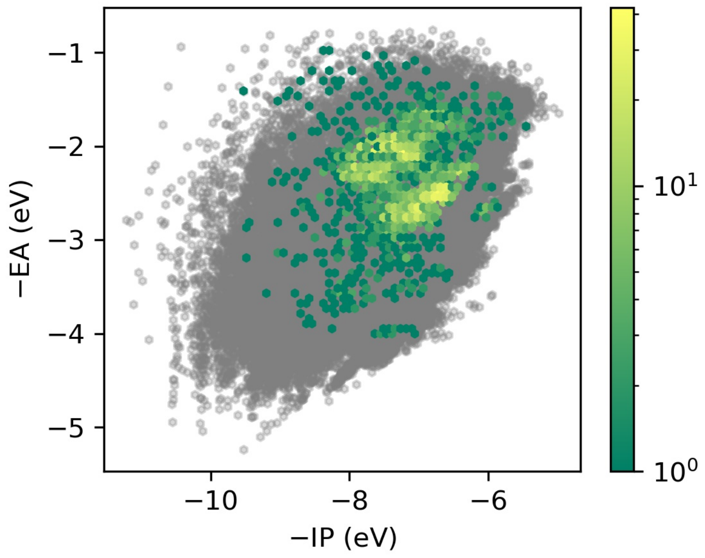

Supplementary Fig. 8 2D histogram of the property space spanned by -IP and -EA for molecules functionalised with one or two OH groups.

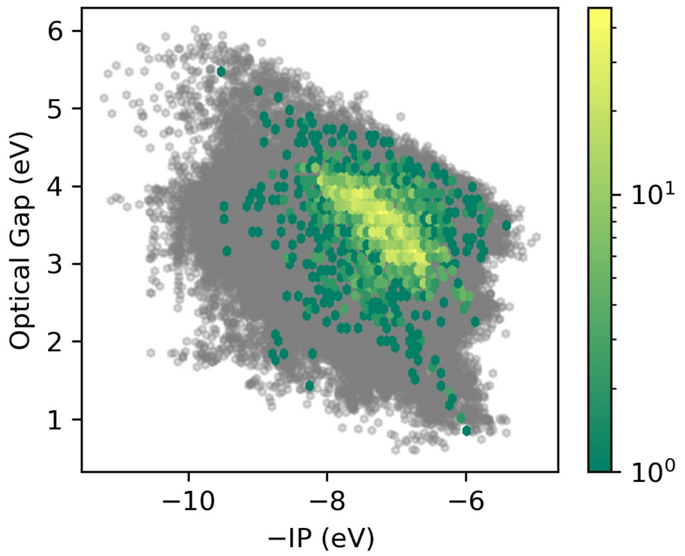

Supplementary Fig. 9 2D histogram of the property space spanned by -IP and the optical gap for molecules functionalised with one or two OH groups.

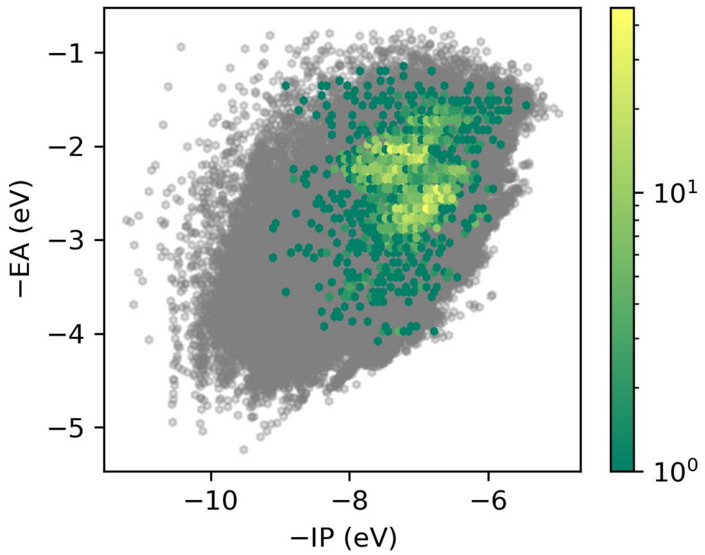

Supplementary Fig. 10 2D histogram of the property space spanned by -IP and -EA for molecules functionalised with one or two OCH_3 groups.

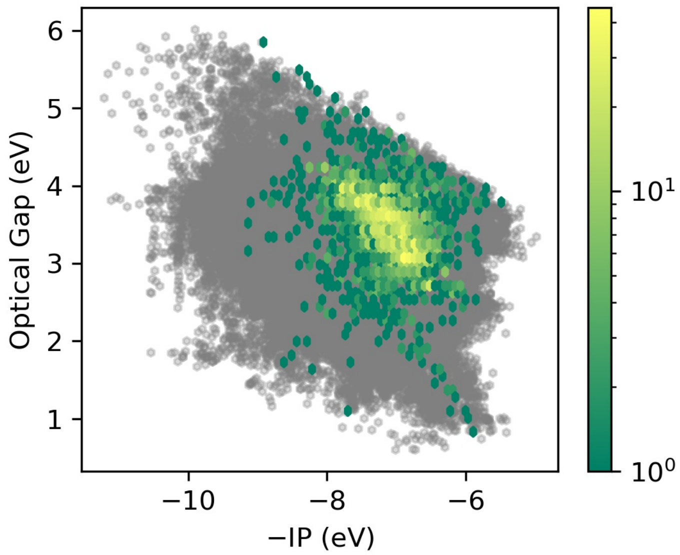

Supplementary Fig. 11 2D histogram of the property space spanned by -IP and the optical gap for molecules functionalised with one or two OCH_3 groups.

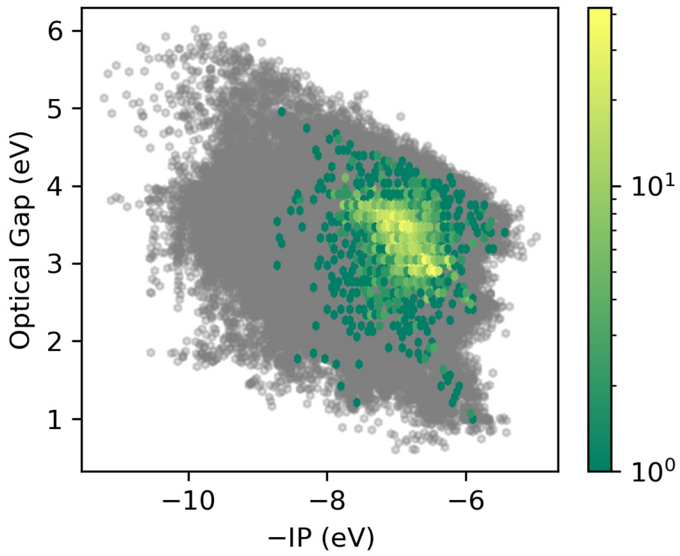

Supplementary Fig. 12 2D histogram of the property space spanned by -IP and -EA for molecules functionalised with one or two SH groups.

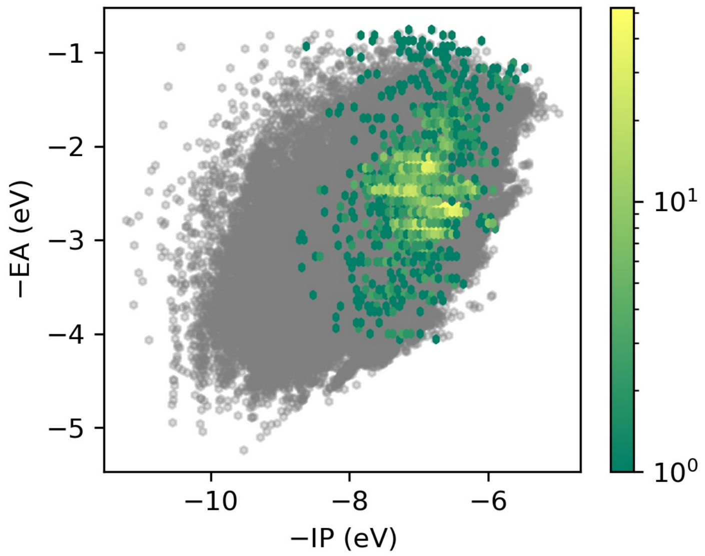

Supplementary Fig. 13 2D histogram of the property space spanned by -IP and the optical gap for molecules functionalised with one or two SH groups.

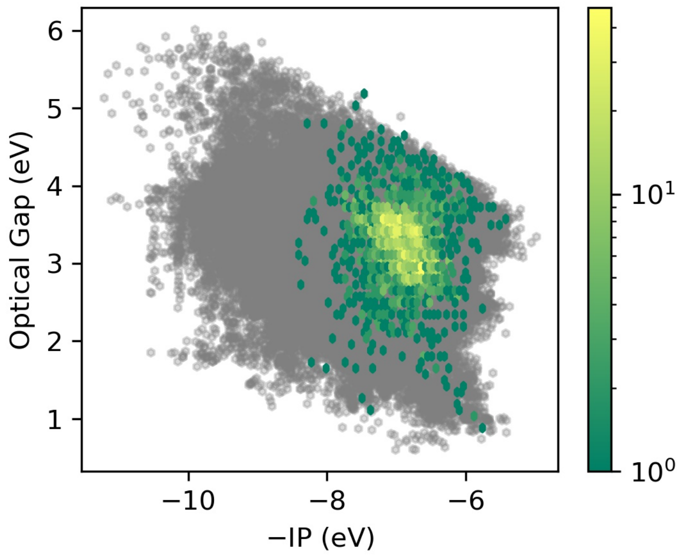

Supplementary Fig. 14 2D histogram of the property space spanned by -IP and -EA for molecules functionalised with one or two SCH_3 groups.

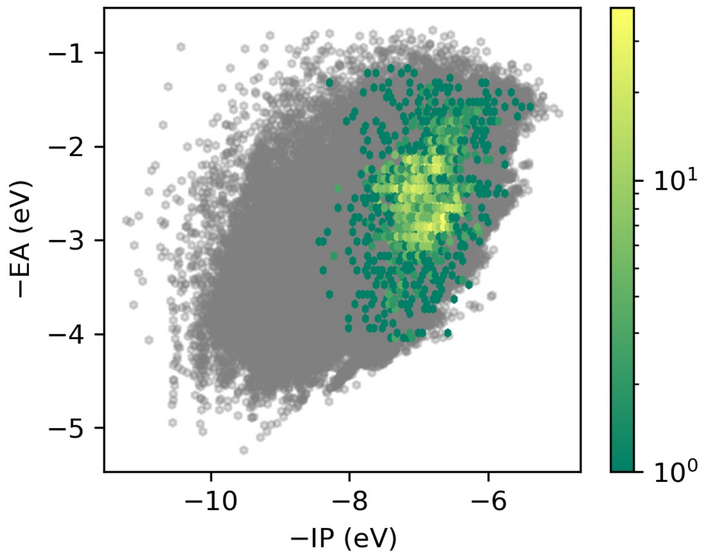

Supplementary Fig. 15 2D histogram of the property space spanned by -IP and the optical gap for molecules functionalised with one or two SCH_3 groups.

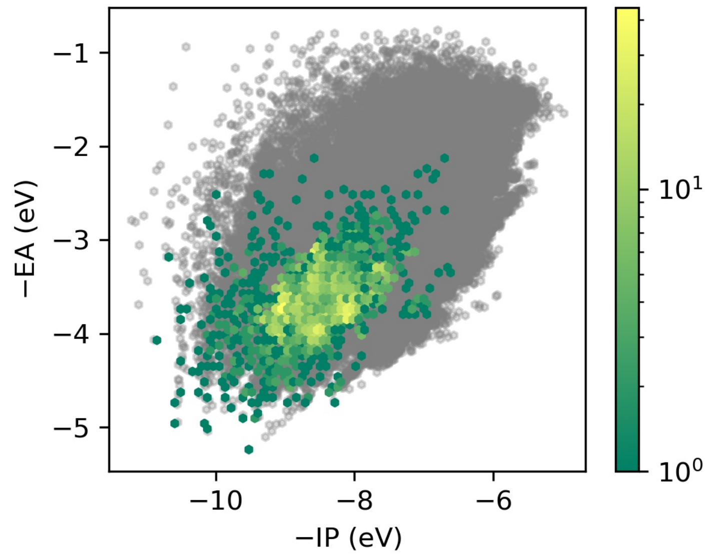

Supplementary Fig. 16 2D histogram of the property space spanned by -IP and -EA for molecules functionalised with one or two NO_2 groups.

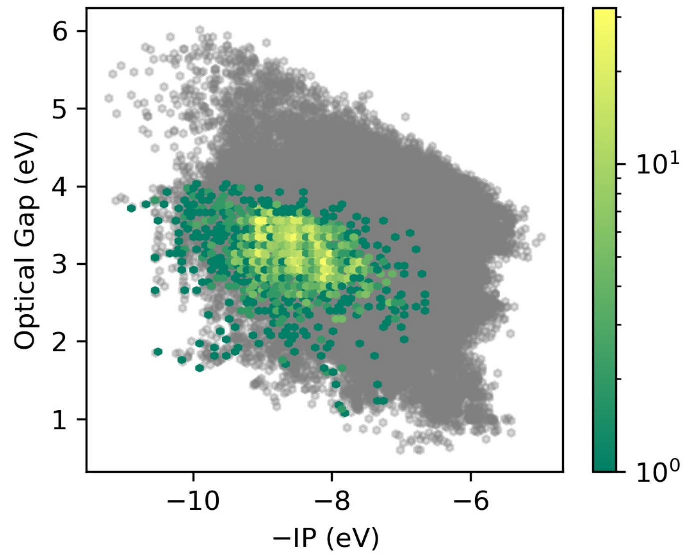

Supplementary Fig. 17 2D histogram of the property space spanned by -IP and the optical gap for molecules functionalised with one or two NO_2 groups.

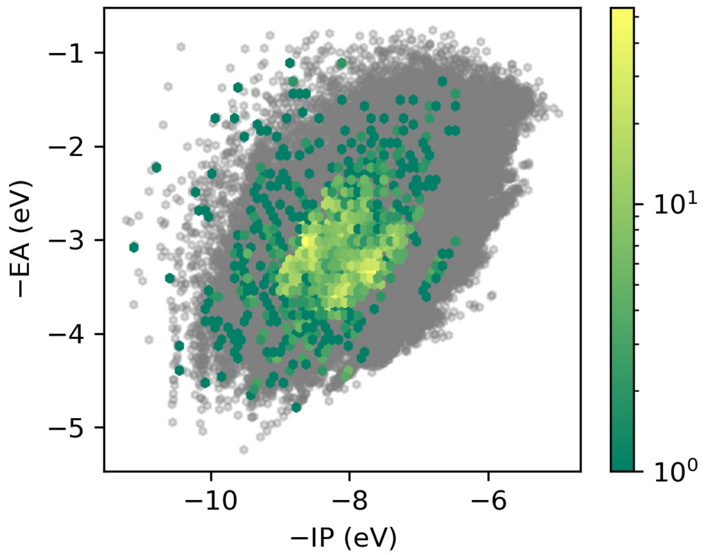

Supplementary Fig. 18 2D histogram of the property space spanned by -IP and -EA for molecules functionalised with one or two CN groups.

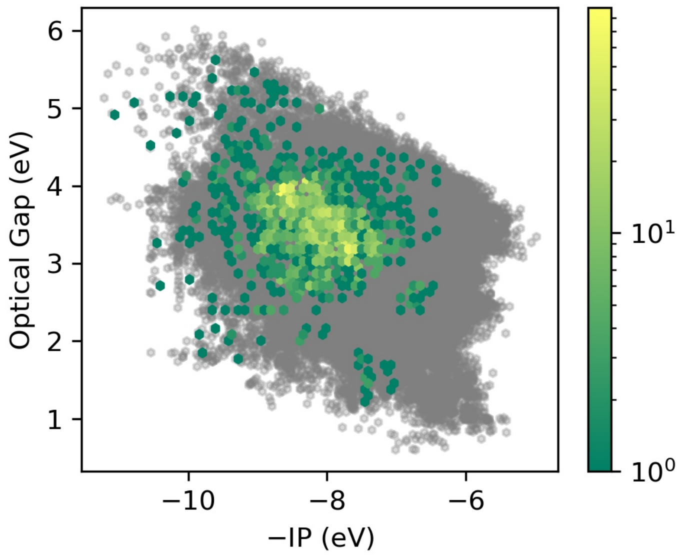

Supplementary Fig. 19 2D histogram of the property space spanned by -IP and the optical gap for molecules functionalised with one or two CN groups.

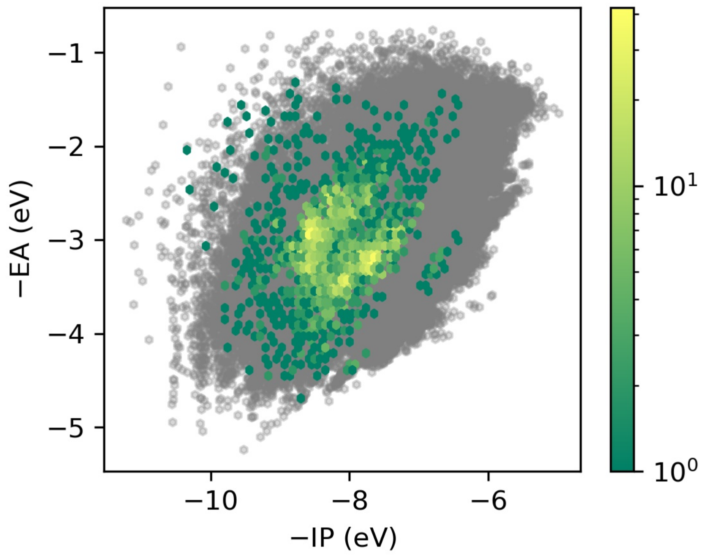

Supplementary Fig. 20 2D histogram of the property space spanned by -IP and -EA for molecules functionalised with one or two SO_3H groups.

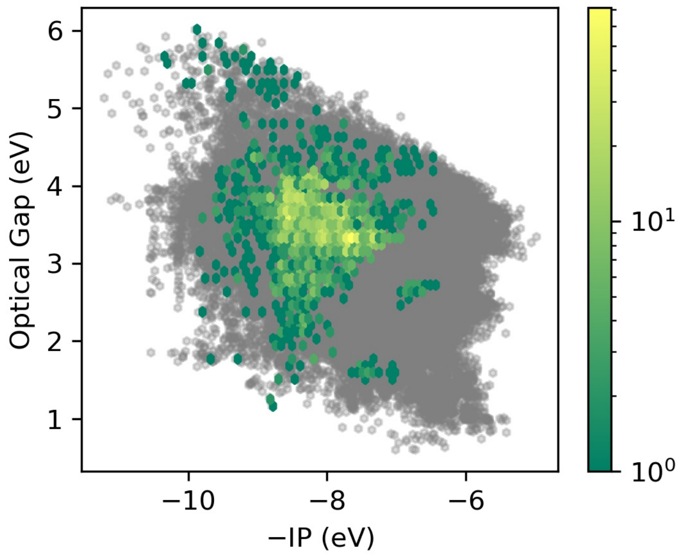

Supplementary Fig. 21 2D histogram of the property space spanned by -IP and the optical gap for molecules functionalised with one or two SO_3H groups.

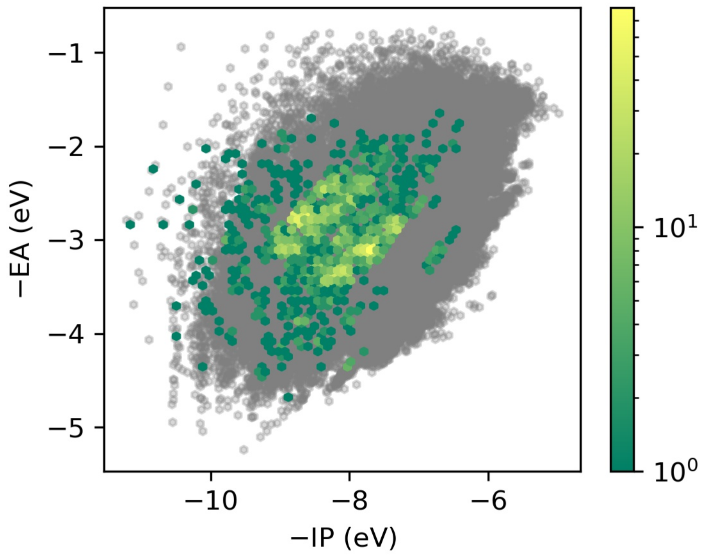

Supplementary Fig. 22 2D histogram of the property space spanned by -IP and -EA for molecules functionalised with one or two CF_3 groups.

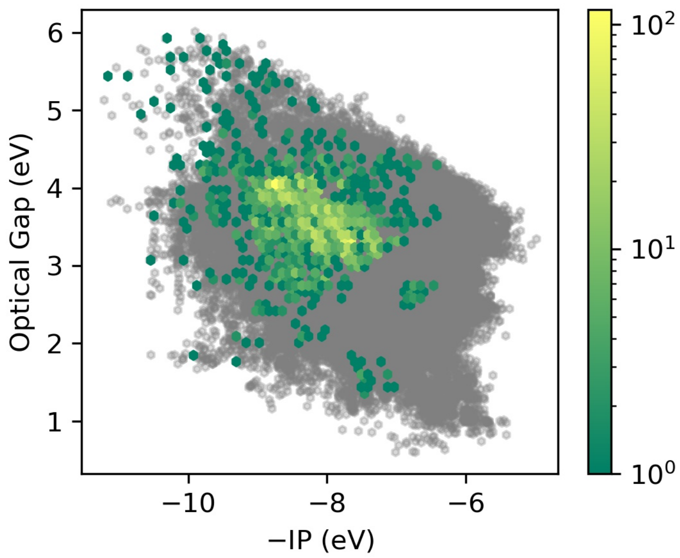

Supplementary Fig. 23 2D histogram of the property space spanned by -IP and the optical gap for molecules functionalised with one or two CF_3 groups.

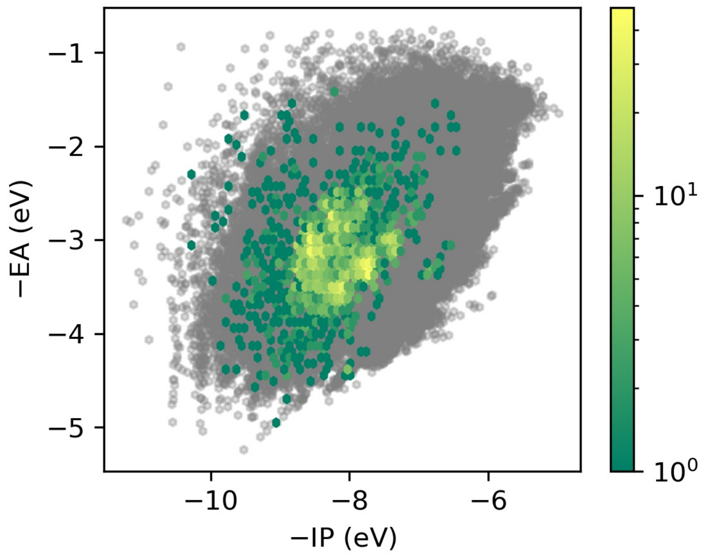

Supplementary Fig. 24 2D histogram of the property space spanned by -IP and -EA for molecules functionalised with one or two COOH groups.

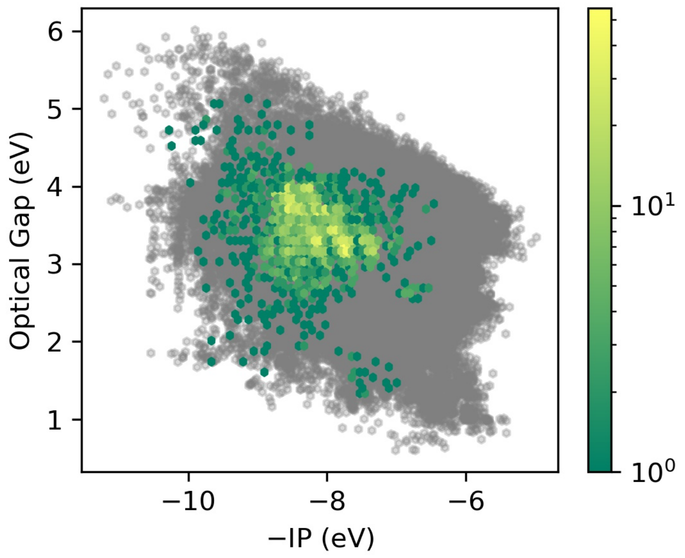

Supplementary Fig. 25 2D histogram of the property space spanned by -IP and the optical gap for molecules functionalised with one or two COOH groups.

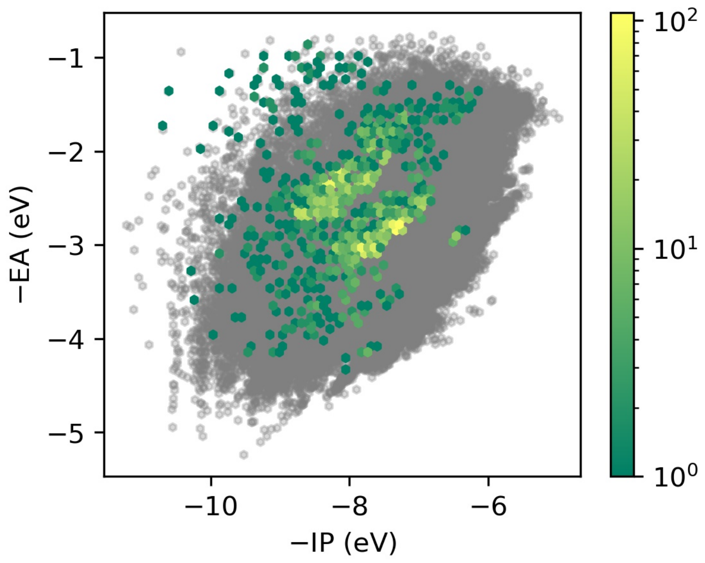

Supplementary Fig. 26 2D histogram of the property space spanned by -IP and -EA for molecules functionalised with one or two fluorine atoms.

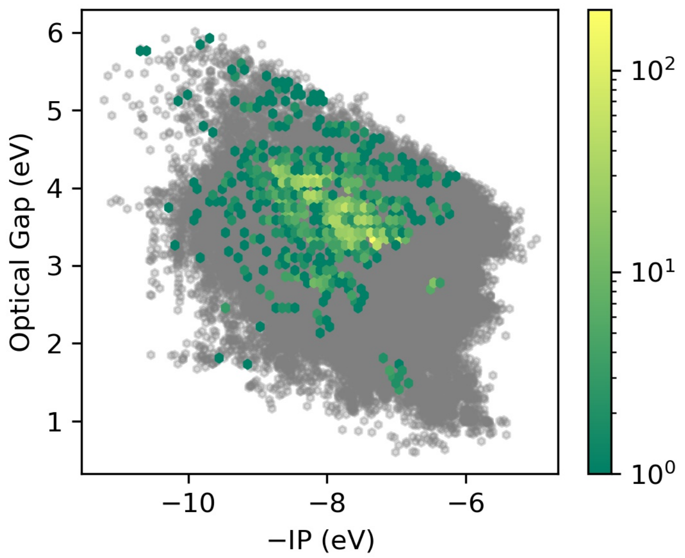

Supplementary Fig. 27 2D histogram of the property space spanned by -IP and the optical gap for molecules functionalised with one or two fluorine atoms.

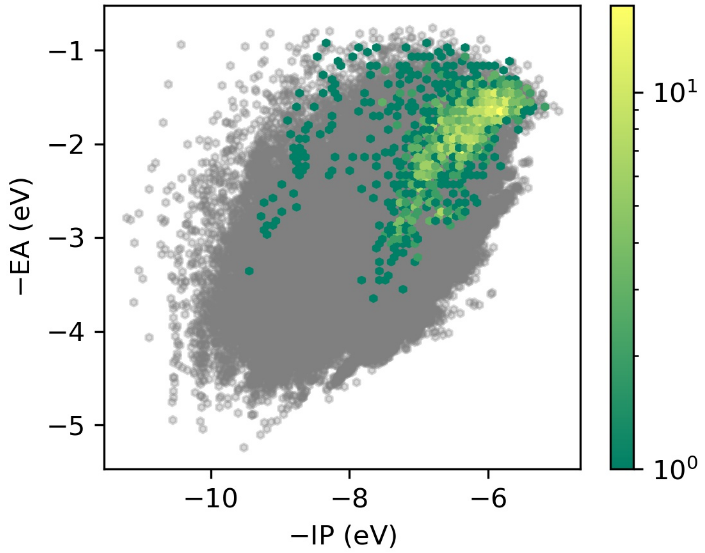

Supplementary Fig. 28 2D histogram of the property space spanned by -IP and -EA for molecules containing [nH](:[cH]):[cH].

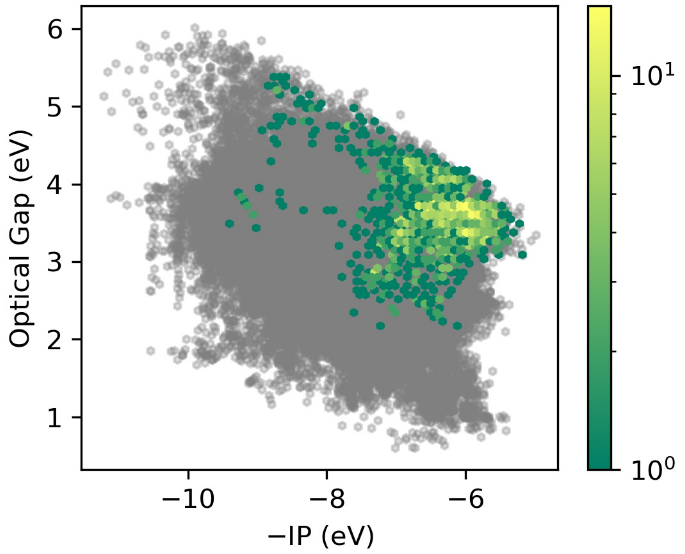

Supplementary Fig. 29 2D histogram of the property space spanned by -IP and the optical gap for molecules containing [nH](:[cH]):[cH].

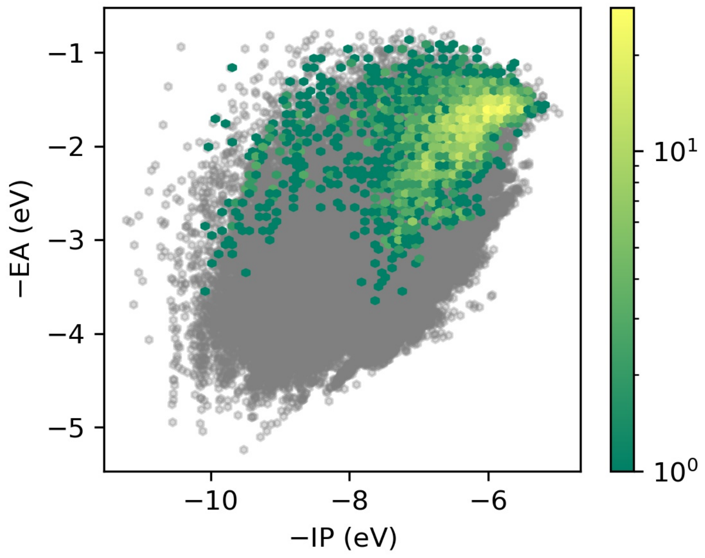

Supplementary Fig. 30 2D histogram of the property space spanned by -IP and -EA for molecules containing [nH](:[cH]):[c].

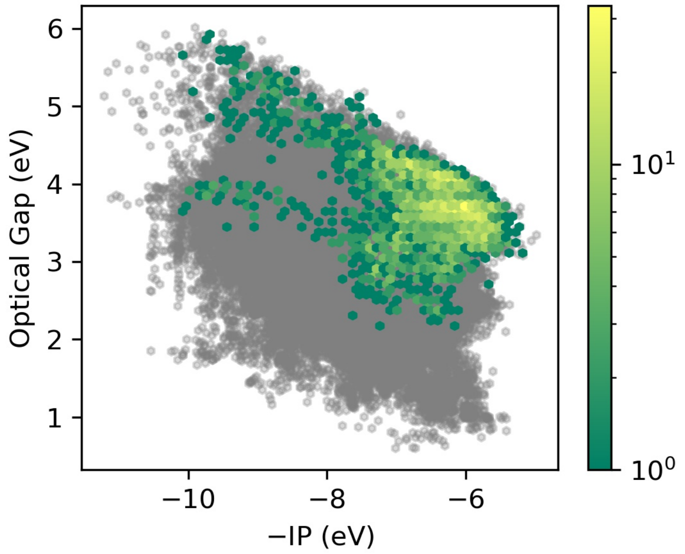

Supplementary Fig. 31 2D histogram of the property space spanned by -IP and the optical gap for molecules containing [nH](:[cH]):[c].

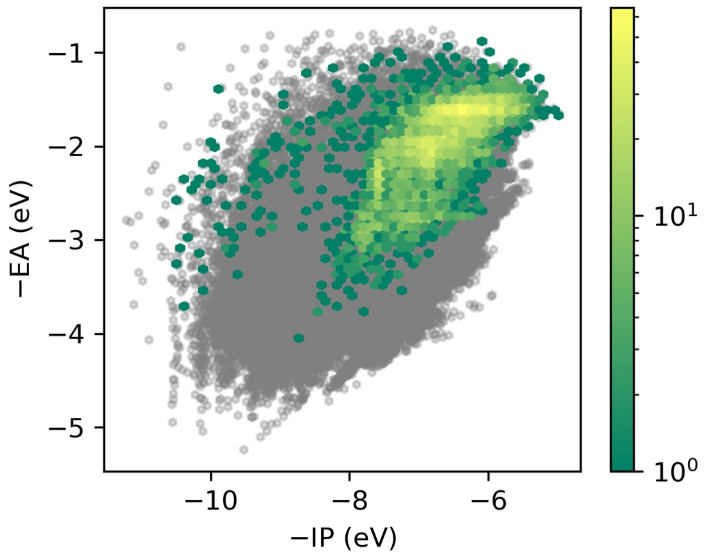

Supplementary Fig. 32 2D histogram of the property space spanned by -IP and -EA for molecules containing [nH](:[c]):[c].

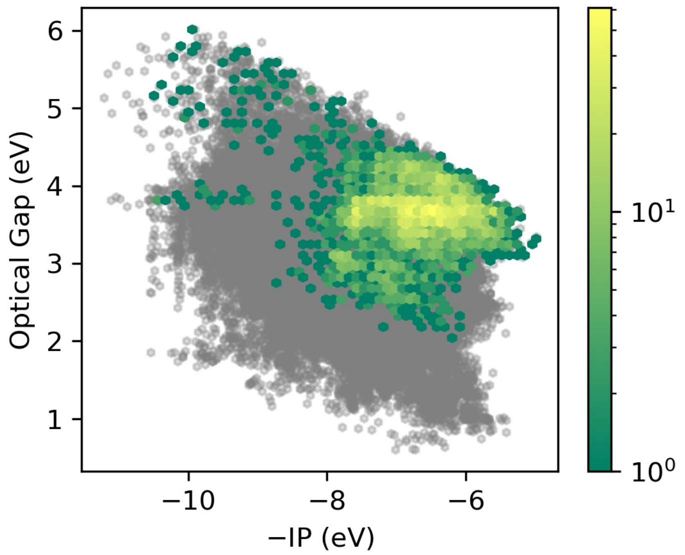

Supplementary Fig. 33 2D histogram of the property space spanned by -IP and the optical gap for molecules containing [nH](:[c]):[c].

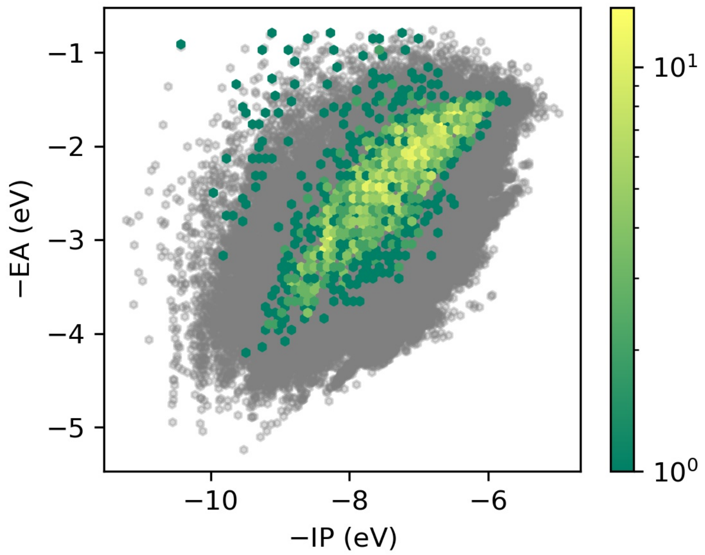

Supplementary Fig. 34 2D histogram of the property space spanned by -IP and -EA for molecules containing [o](:[cH]):[cH].

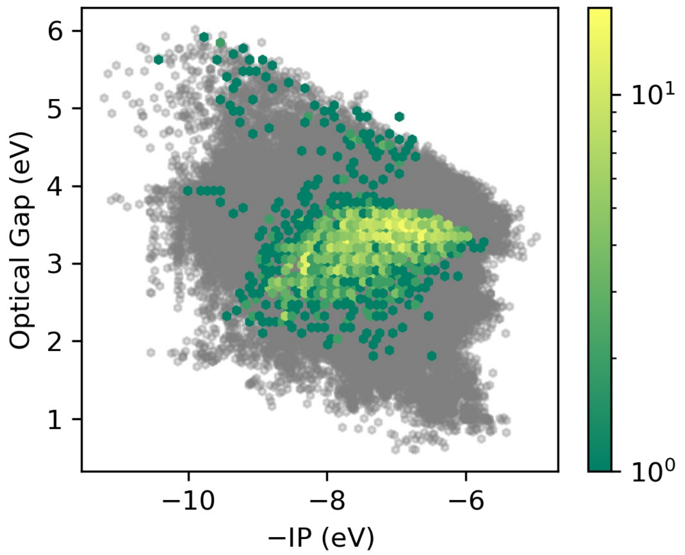

Supplementary Fig. 35 2D histogram of the property space spanned by -IP and the optical gap for molecules containing [o](:[cH]):[cH].

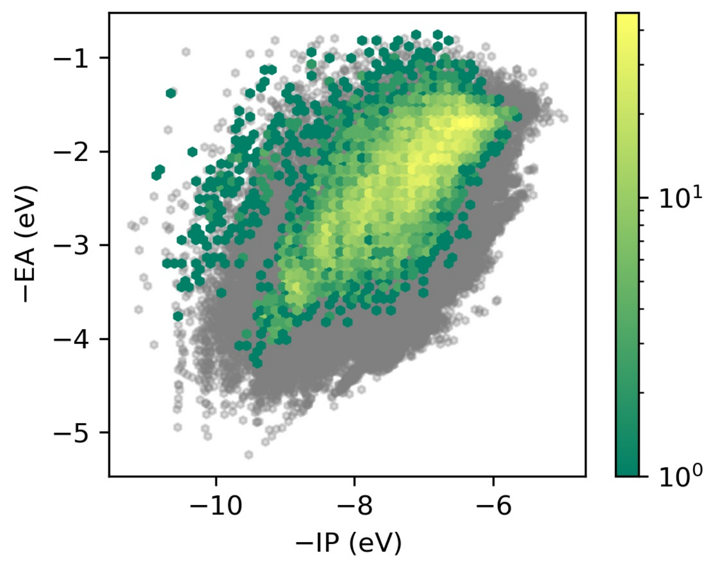

Supplementary Fig. 36 2D histogram of the property space spanned by -IP and -EA for molecules containing [o](:[cH]):[c].

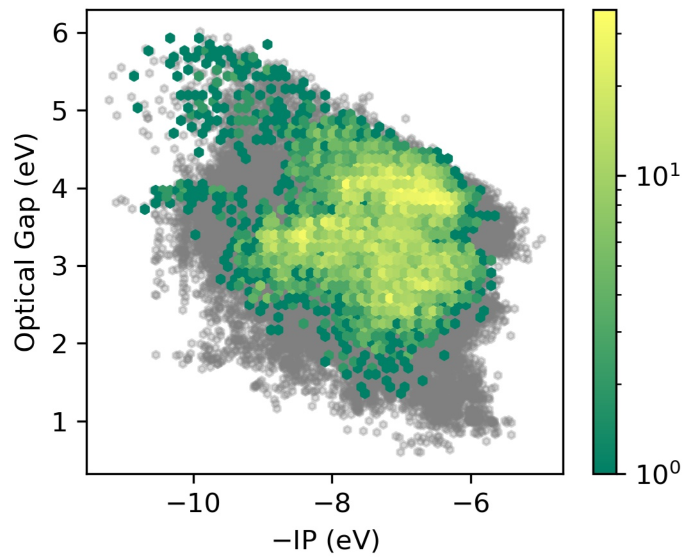

Supplementary Fig. 37 2D histogram of the property space spanned by -IP and the optical gap for molecules containing [o](:[cH]):[c].

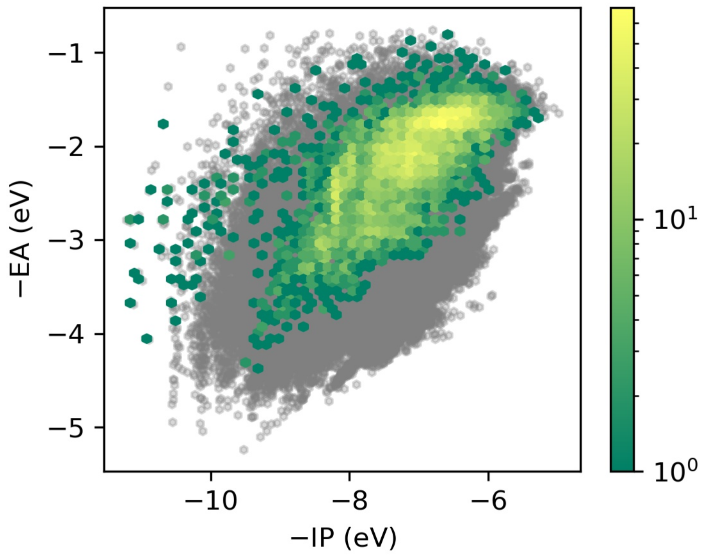

Supplementary Fig. 38 2D histogram of the property space spanned by -IP and -EA for molecules containing [o](:[c]):[c].

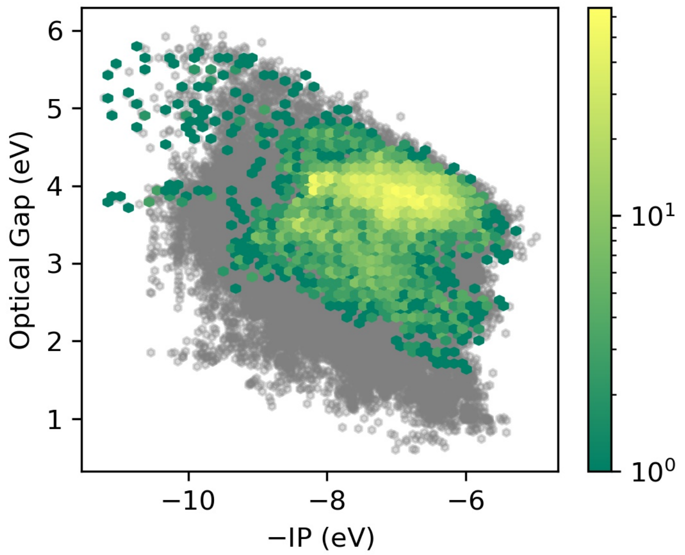

Supplementary Fig. 39 2D histogram of the property space spanned by -IP and the optical gap for molecules containing [o](:[c]):[c].

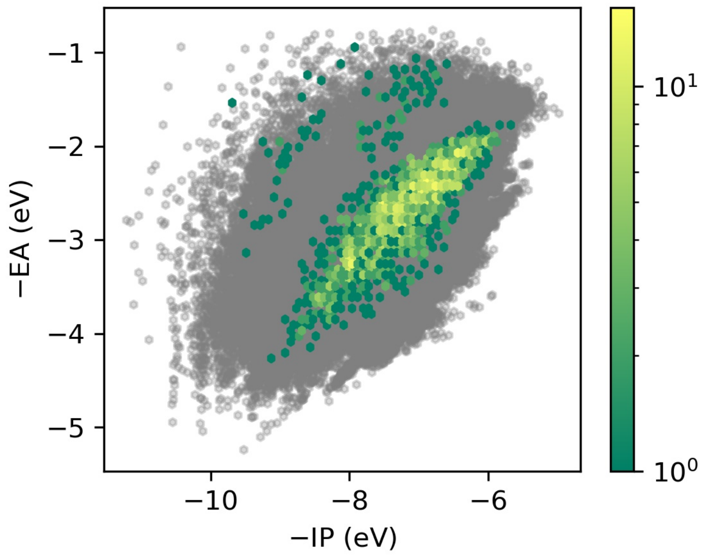

Supplementary Fig. 40 2D histogram of the property space spanned by -IP and -EA for molecules containing [s](:[cH]):[cH].

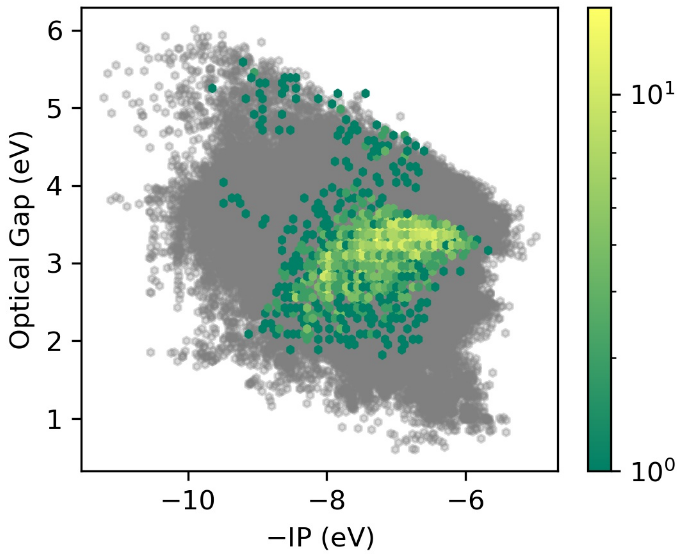

Supplementary Fig. 41 2D histogram of the property space spanned by -IP and the optical gap for molecules containing [s](:[cH]):[cH].

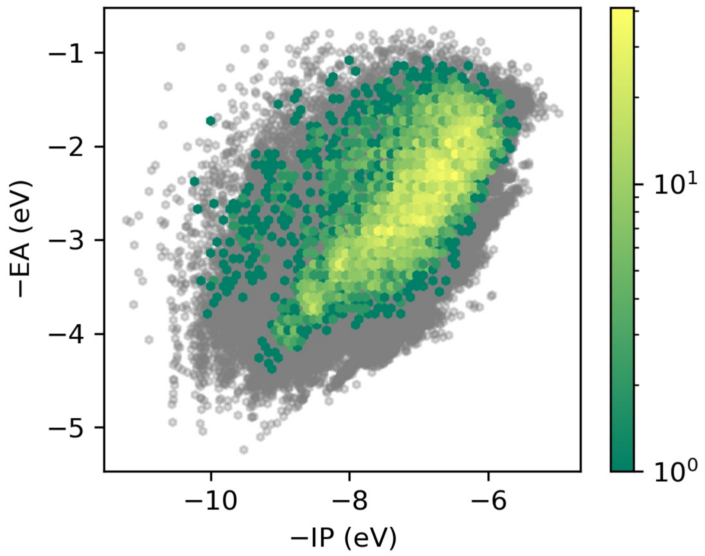

Supplementary Fig. 42 2D histogram of the property space spanned by -IP and -EA for molecules containing [s](:[cH]):[c].

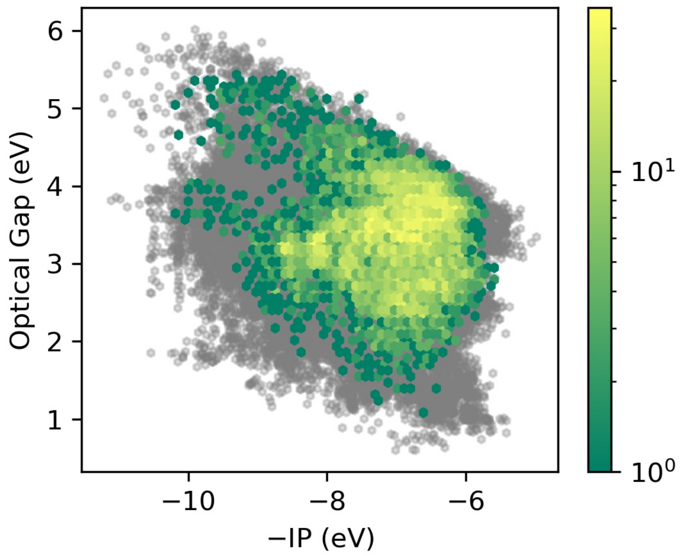

Supplementary Fig. 43 2D histogram of the property space spanned by -IP and the optical gap for molecules containing [s](:[cH]):[c].

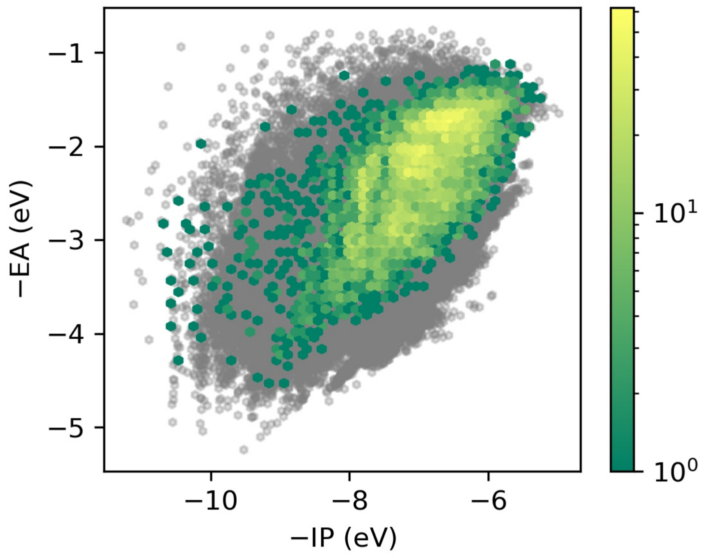

Supplementary Fig. 44 2D histogram of the property space spanned by -IP and -EA for molecules containing [s](:[c]):[c].

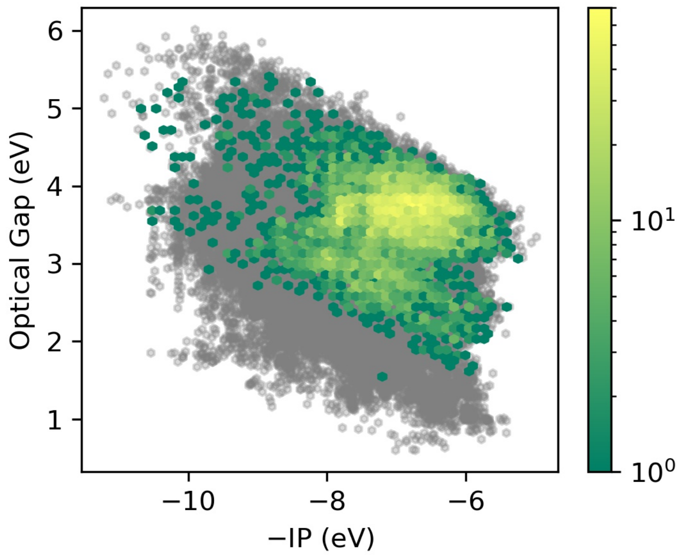

Supplementary Fig. 45 2D histogram of the property space spanned by -IP and the optical gap for molecules containing [s](:[c]):[c].

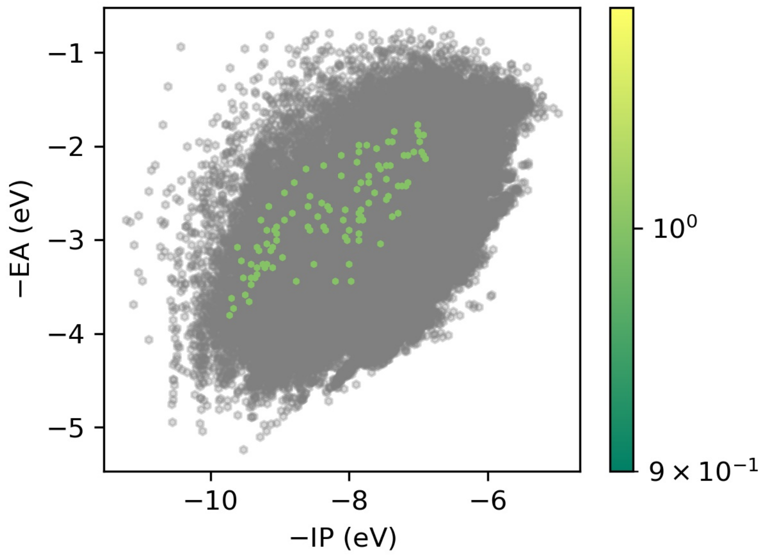

Supplementary Fig. 46 2D histogram of the property space spanned by -IP and -EA for molecules containing [S](-[CH])(-[CH])(=[O])=[O].

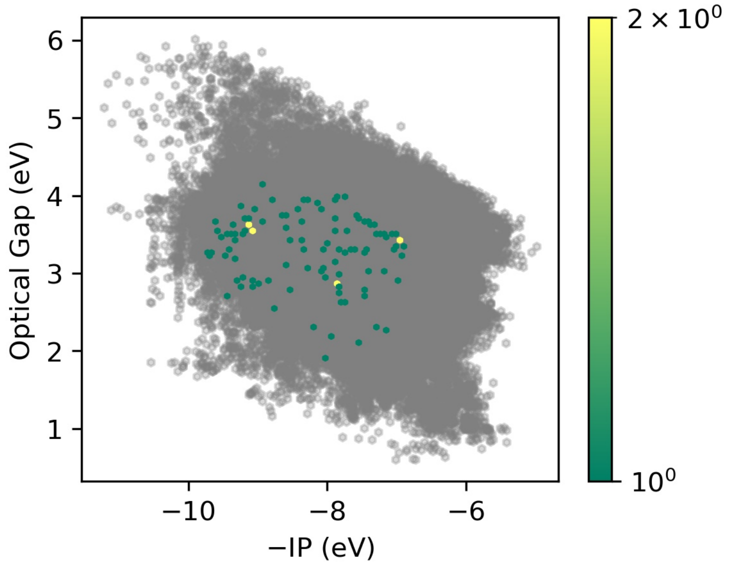

Supplementary Fig. 47 2D histogram of the property space spanned by -IP and the optical gap for molecules containing [S](-[CH])(-[CH])(=[O])=[O].

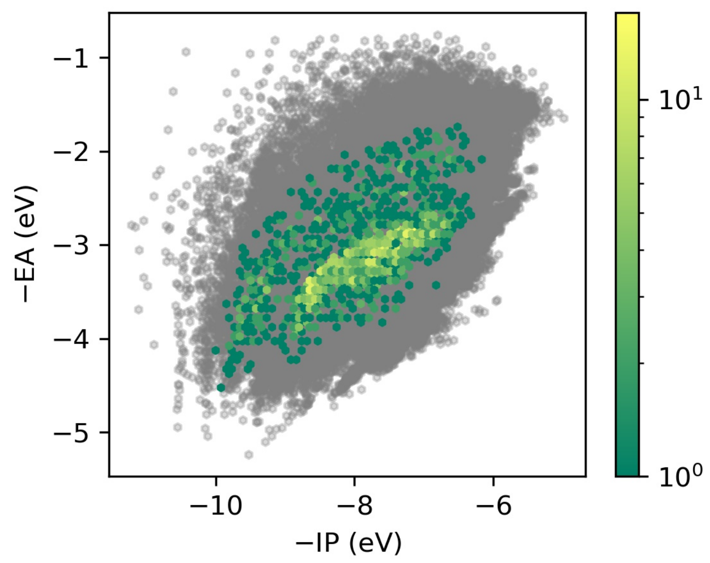

Supplementary Fig. 48 2D histogram of the property space spanned by -IP and -EA for molecules containing [S](-[CH])(-[C])(=[O])=[O].

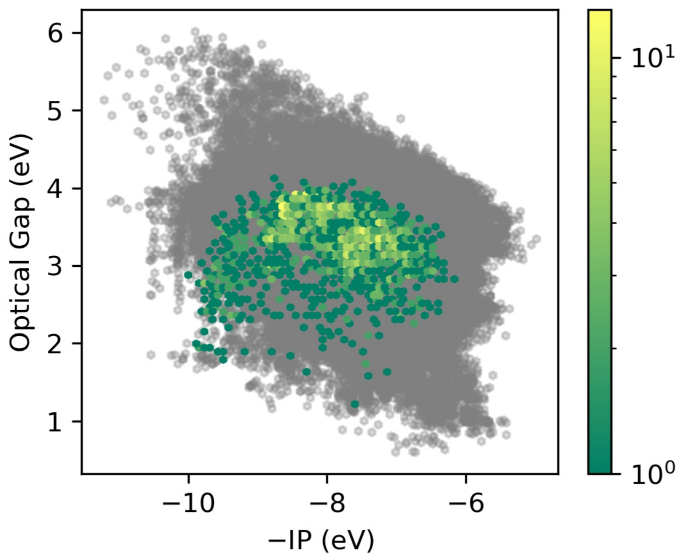

Supplementary Fig. 49 2D histogram of the property space spanned by -IP and the optical gap for molecules containing [S](-[CH])(-[C])(=[O])=[O].

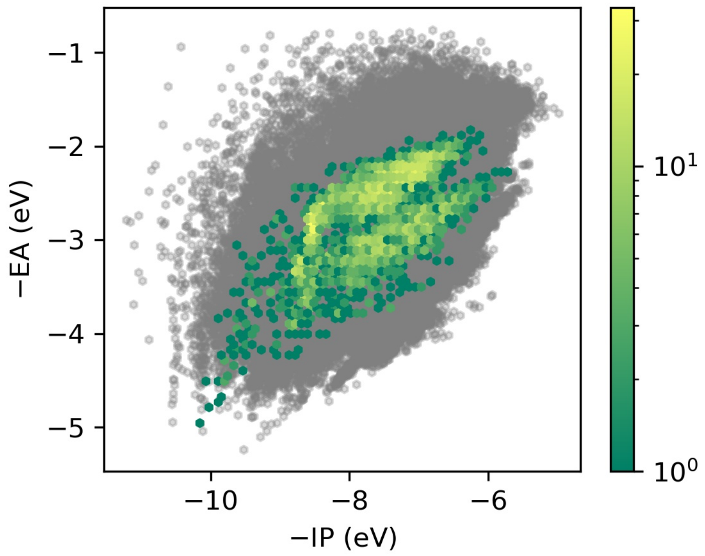

Supplementary Fig. 50 2D histogram of the property space spanned by -IP and -EA for molecules containing [S](-[C])(-[C])(=[O])=[O].

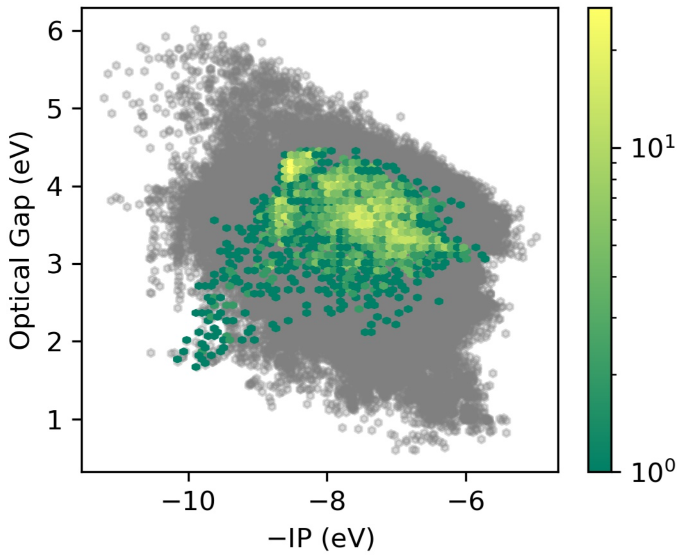

Supplementary Fig. 51 2D histogram of the property space spanned by -IP and the optical gap for molecules containing [S](-[C])(-[C])(=[O])=[O].

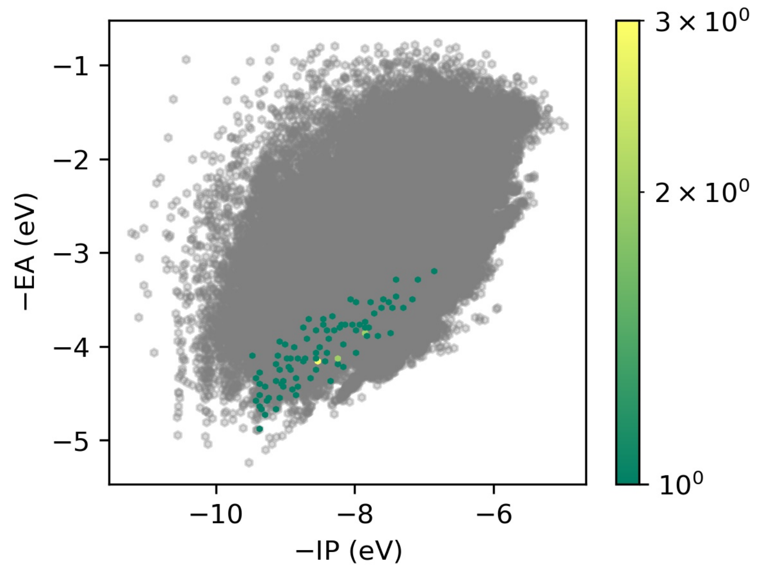

Supplementary Fig. 52 2D histogram of the property space spanned by -IP and -EA for molecules containing [C](-[CH])(-[CH])=[O] or [C](-[cH])(-[cH])=[O].

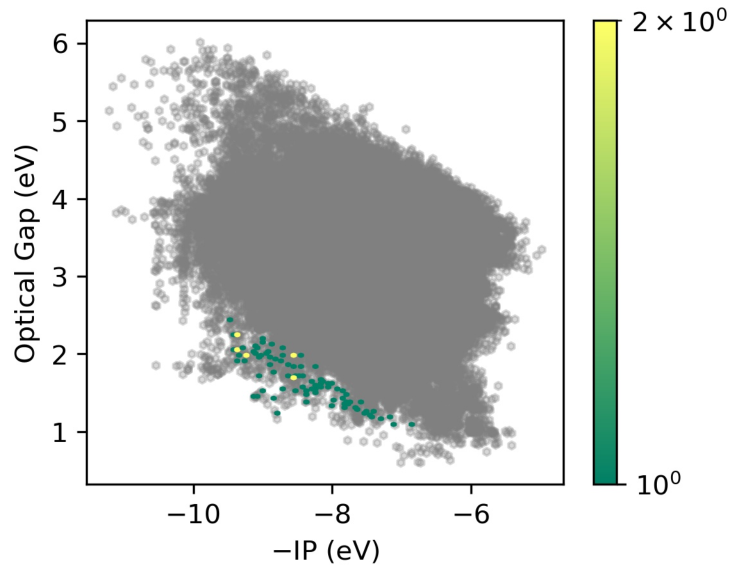

Supplementary Fig. 53 2D histogram of the property space spanned by -IP and the optical gap for molecules containing [C](-[CH])(-[CH])=[O] or [C](-[cH])(-[cH])=[O].

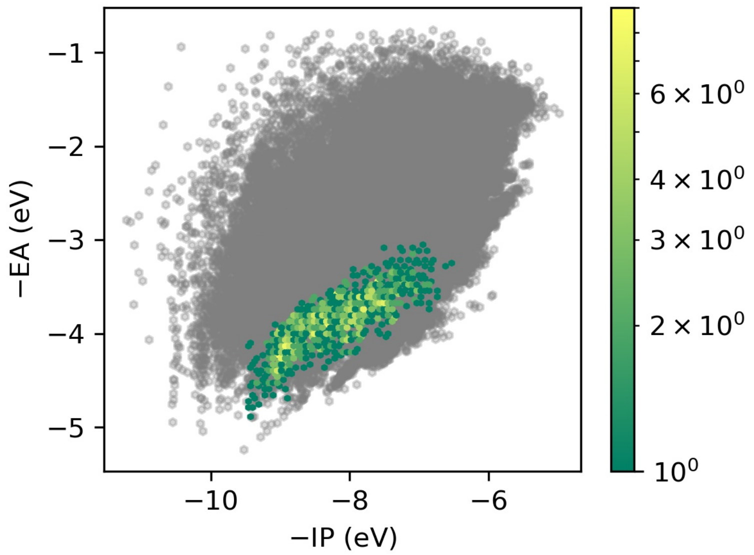

Supplementary Fig. 54 2D histogram of the property space spanned by -IP and -EA for molecules containing [C](-[CH])(-[C])=[O] or [C](-[cH])(-[c])=[O].

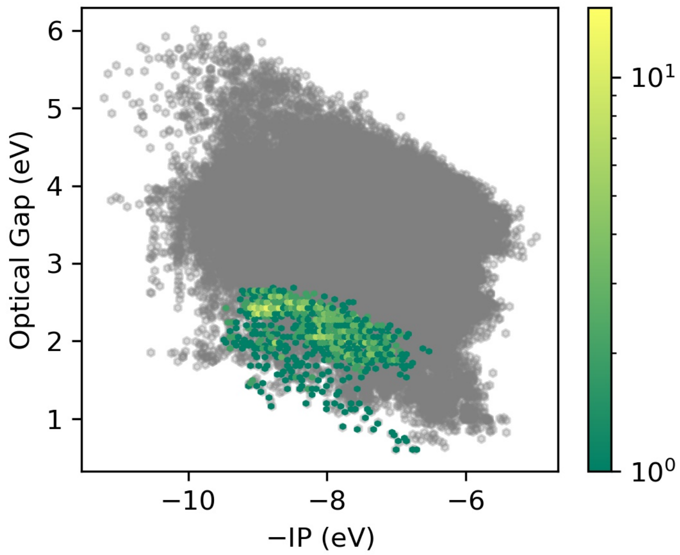

Supplementary Fig. 55 2D histogram of the property space spanned by -IP and the optical gap for molecules containing [C](-[CH])(-[C])=[O] or [C](-[cH])(-[c])=[O].

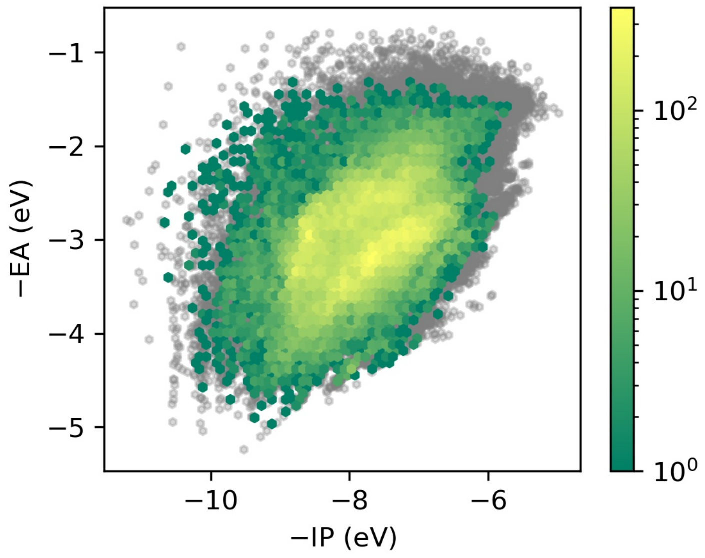

Supplementary Fig. 56 2D histogram of the property space spanned by -IP and -EA for molecules containing [C](-[C])(-[C])=[O] or [C](-[c])(-[c])=[O].

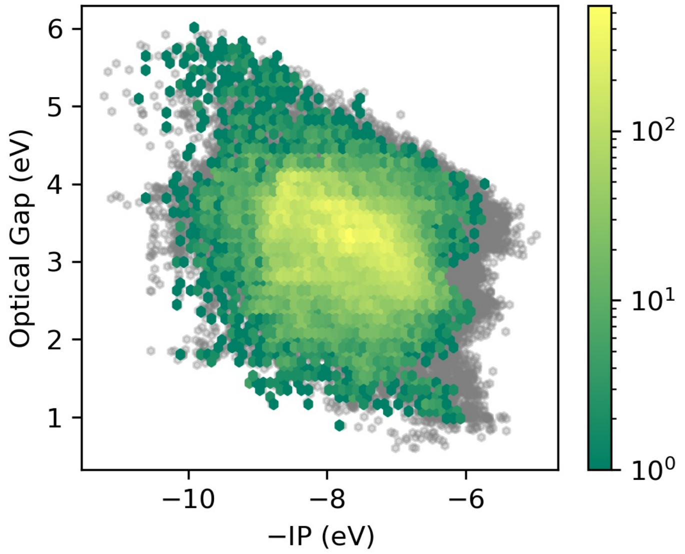

Supplementary Fig. 57 2D histogram of the property space spanned by -IP and the optical gap for molecules containing [C](-[C])(-[C])=[O] or [C](-[c])(-[c])=[O].

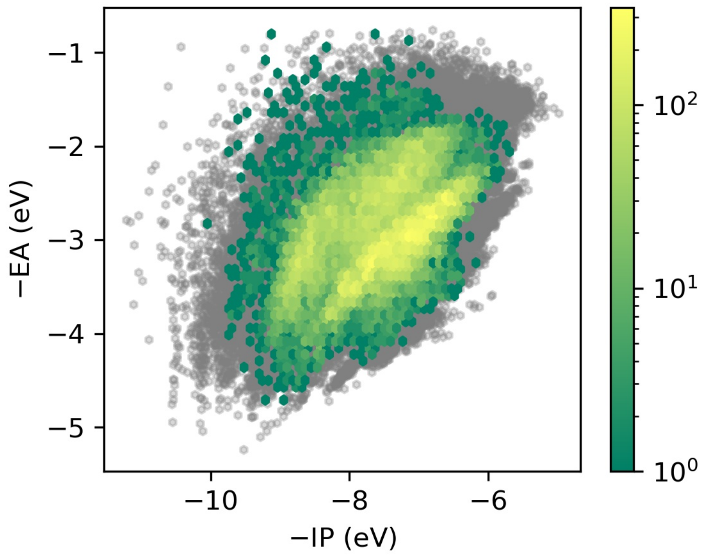

Supplementary Fig. 58 2D histogram of the property space spanned by -IP and -EA for molecules containing [n](:[cH]):[cH].

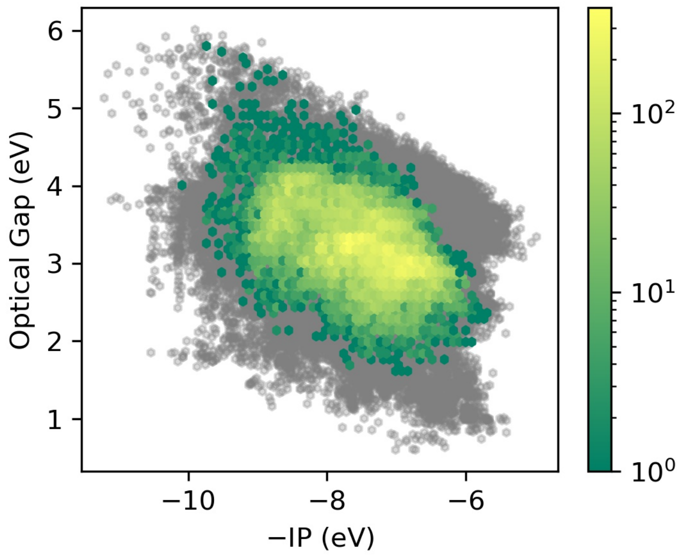

Supplementary Fig. 59 2D histogram of the property space spanned by -IP and the optical gap for molecules containing [n](:[cH]):[cH].

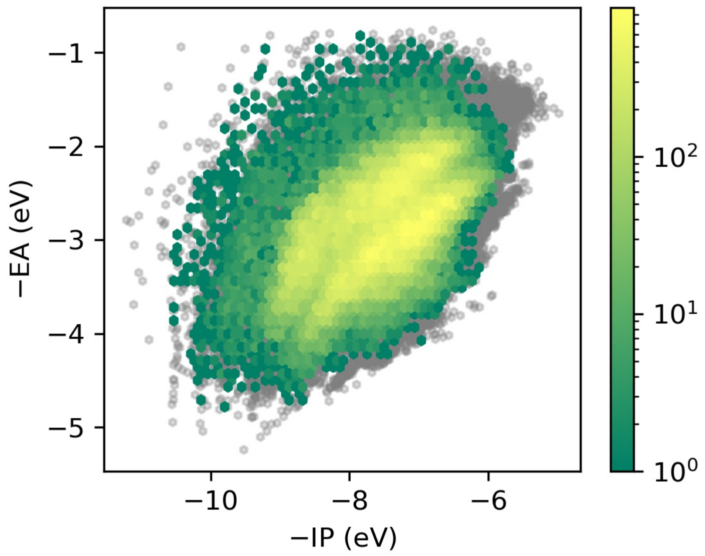

Supplementary Fig. 60 2D histogram of the property space spanned by -IP and -EA for molecules containing [n](:[cH]):[c].

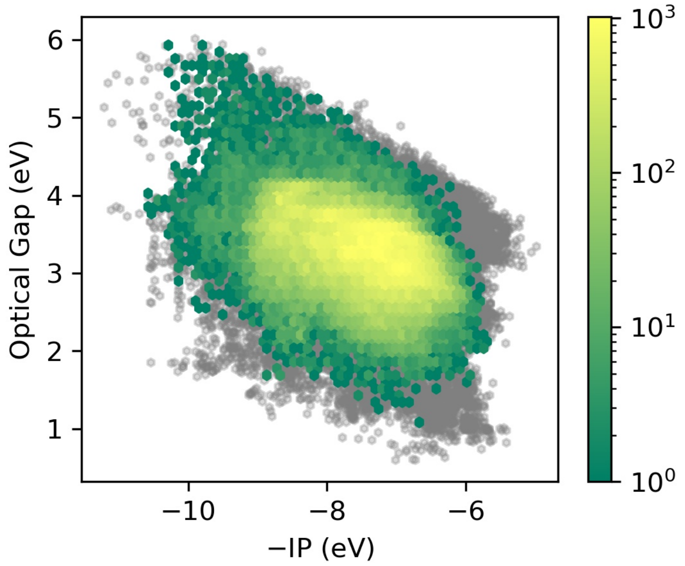

Supplementary Fig. 61 2D histogram of the property space spanned by -IP and the optical gap for molecules containing [n](:[cH]):[c].

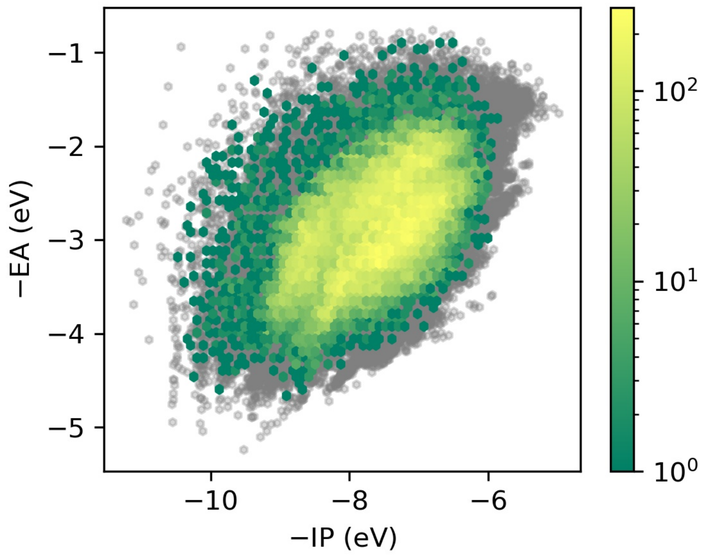

Supplementary Fig. 62 2D histogram of the property space spanned by -IP and -EA for molecules containing [n](:[c]):[c].

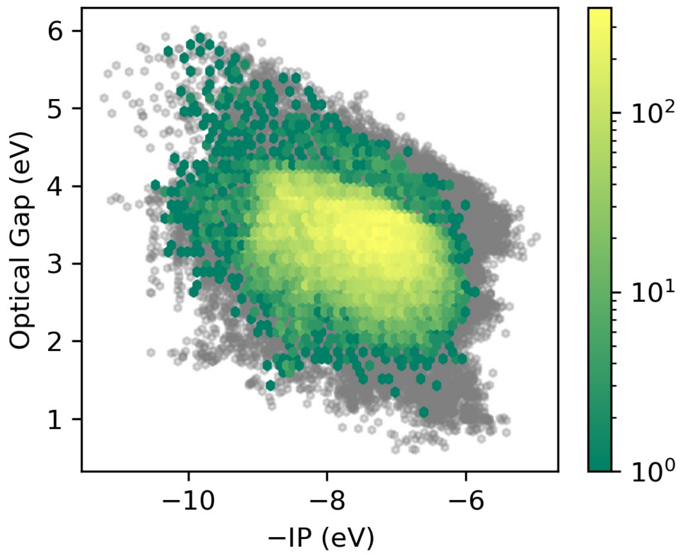

Supplementary Fig. 63 2D histogram of the property space spanned by -IP and the optical gap for molecules containing [n](:[c]):[c].

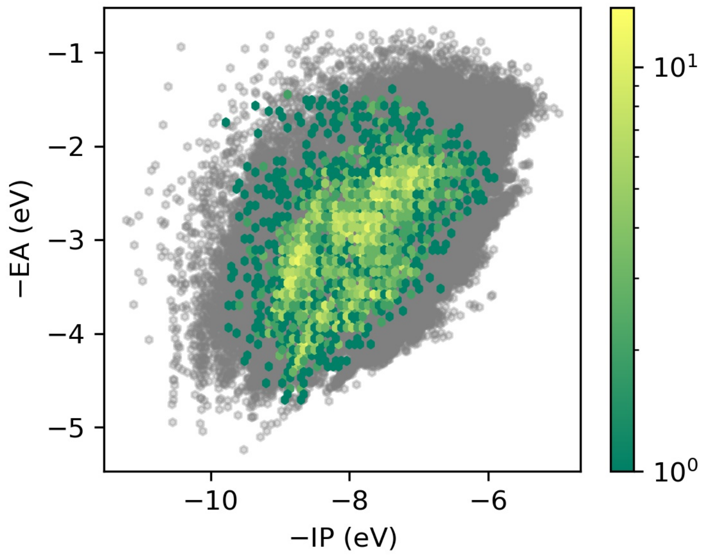

Supplementary Fig. 64 2D histogram of the property space spanned by -IP and -EA for molecules containing [n](:[n]):[cH].

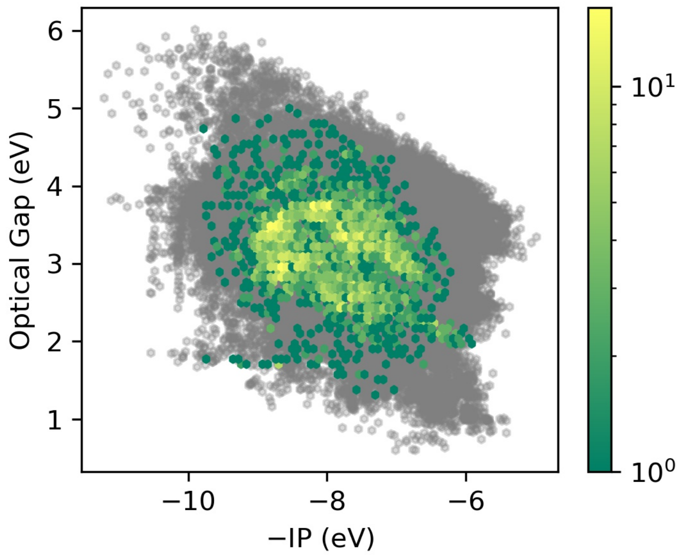

Supplementary Fig. 65 2D histogram of the property space spanned by -IP and the optical gap for molecules containing [n](:[n]):[cH].

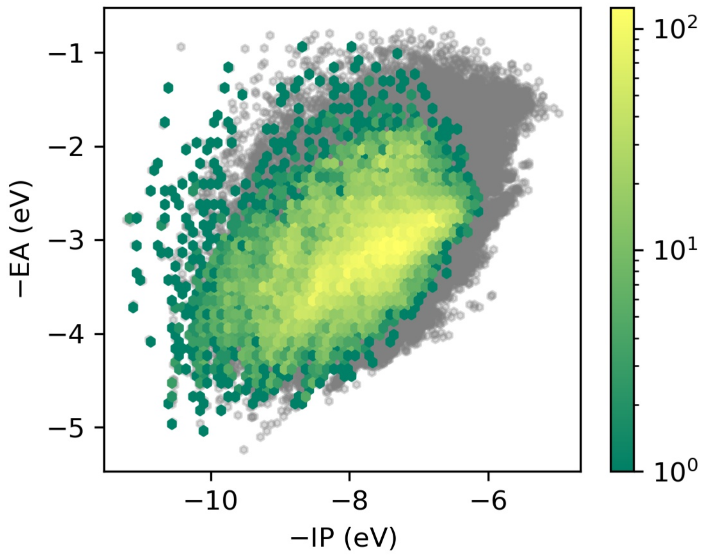

Supplementary Fig. 66 2D histogram of the property space spanned by -IP and -EA for molecules containing [n](:[n]):[c].

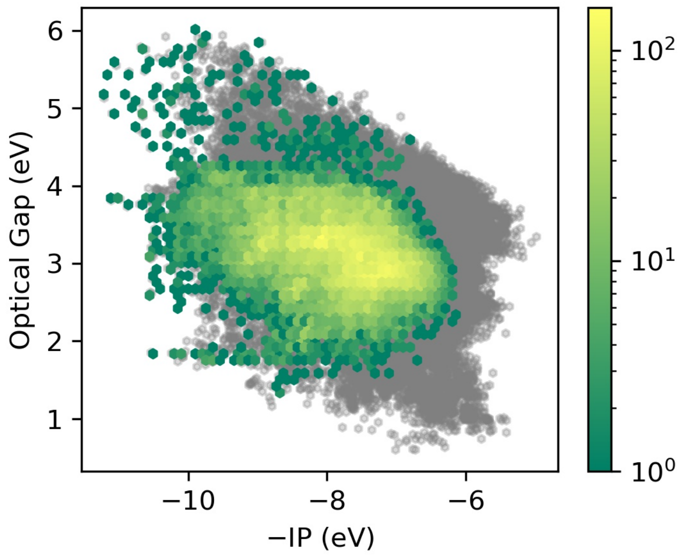

Supplementary Fig. 67 2D histogram of the property space spanned by -IP and the optical gap for molecules containing [n](:[n]):[c].

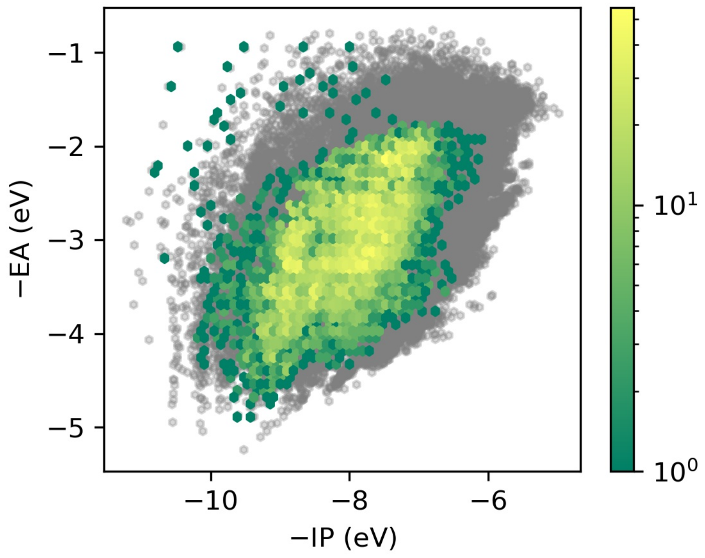

Supplementary Fig. 68 2D histogram of the property space spanned by -IP and -EA for molecules containing [cH](:[n]):[n].

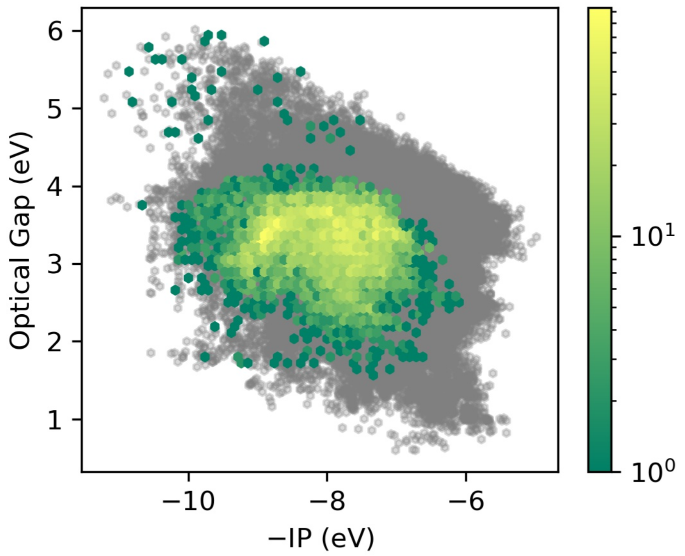

Supplementary Fig. 69 2D histogram of the property space spanned by -IP and the optical gap for molecules containing [cH](:[n]):[n].

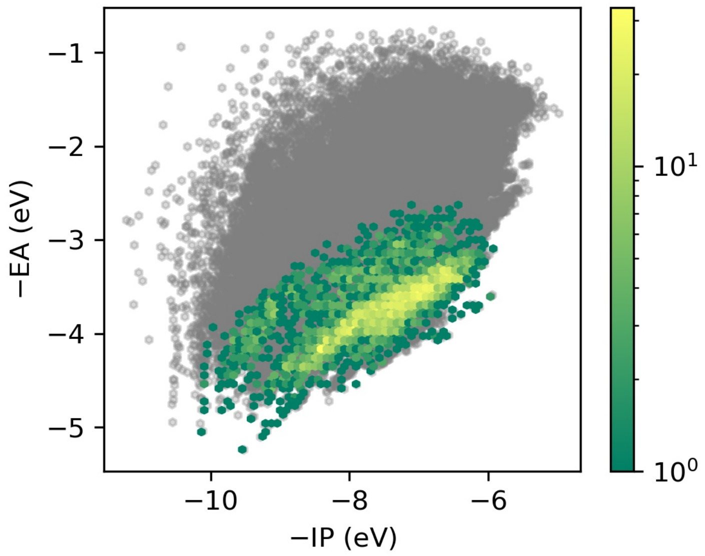

Supplementary Fig. 70 2D histogram of the property space spanned by -IP and -EA for molecules containing [s](:[n]):[n].

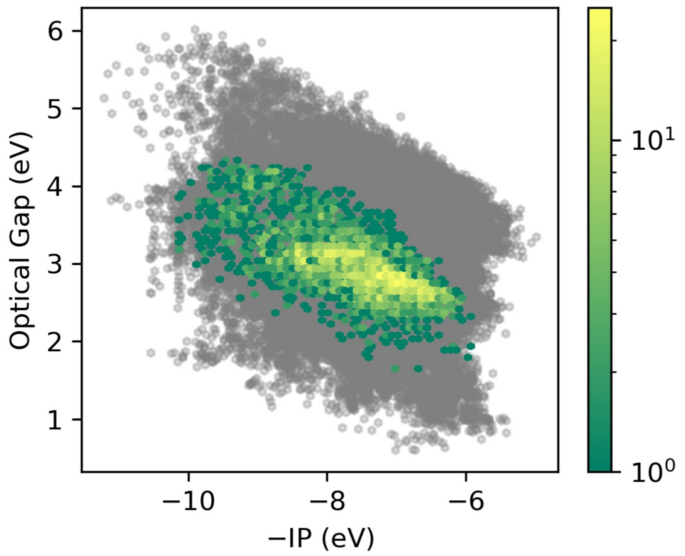

Supplementary Fig. 71 2D histogram of the property space spanned by -IP and the optical gap for molecules containing [s](:[n]):[n].

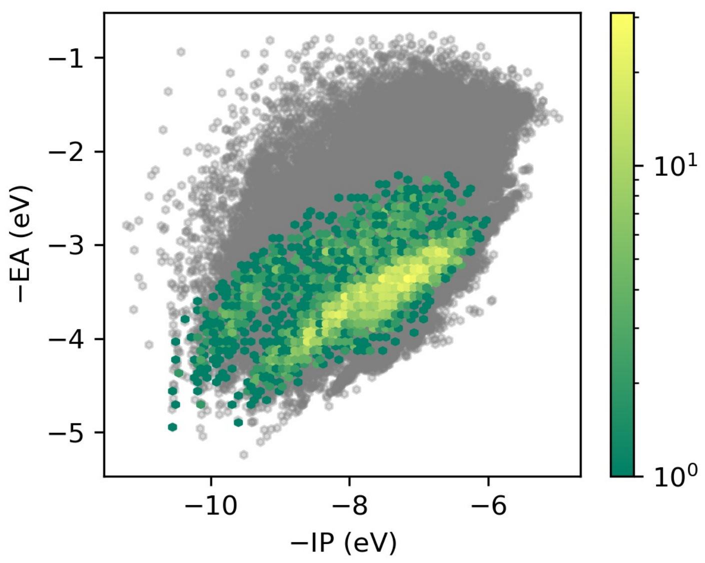

Supplementary Fig. 72 2D histogram of the property space spanned by -IP and -EA for molecules containing [o](:[n]):[n].

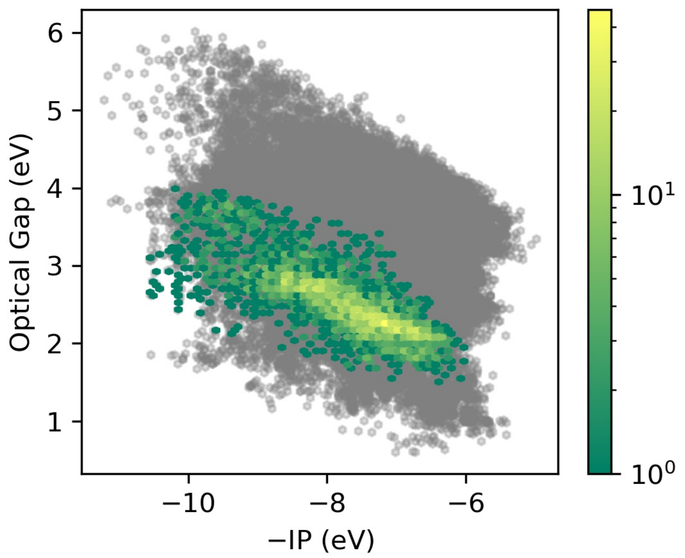

Supplementary Fig. 73 2D histogram of the property space spanned by -IP and the optical gap for molecules containing [o](:[n]):[n].

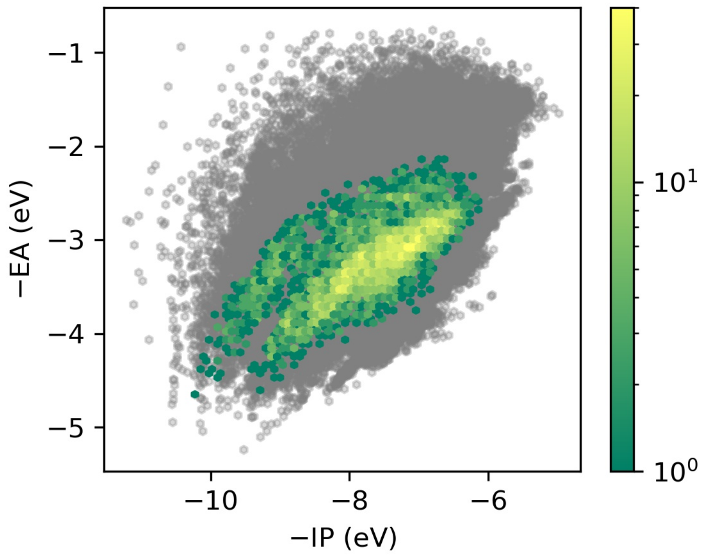

Supplementary Fig. 74 2D histogram of the property space spanned by -IP and -EA for molecules containing [n](:[n]):[s].

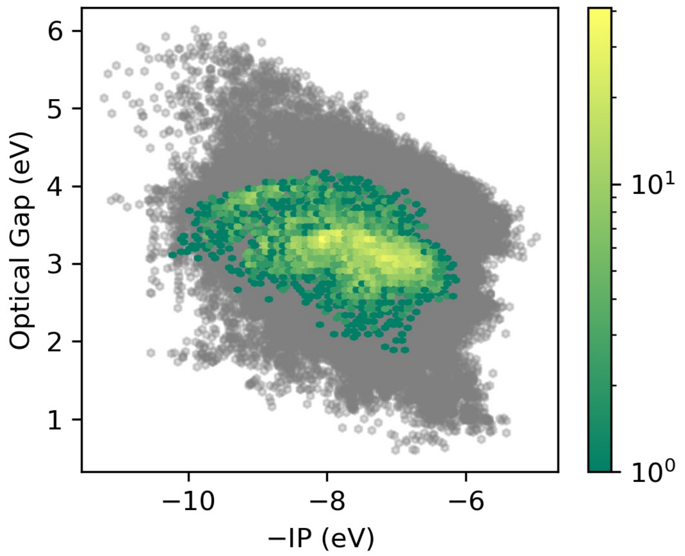

Supplementary Fig. 75 2D histogram of the property space spanned by -IP and the optical gap for molecules containing [n](:[n]):[s].

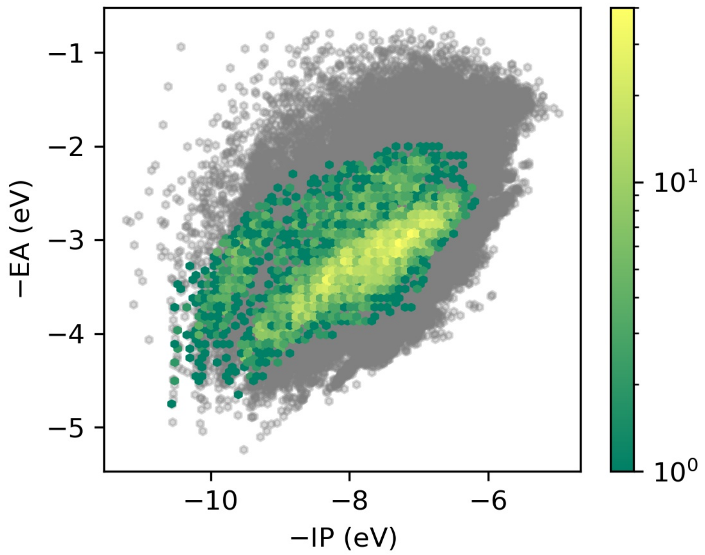

Supplementary Fig. 76 2D histogram of the property space spanned by -IP and -EA for molecules containing [n](:[n]):[o].

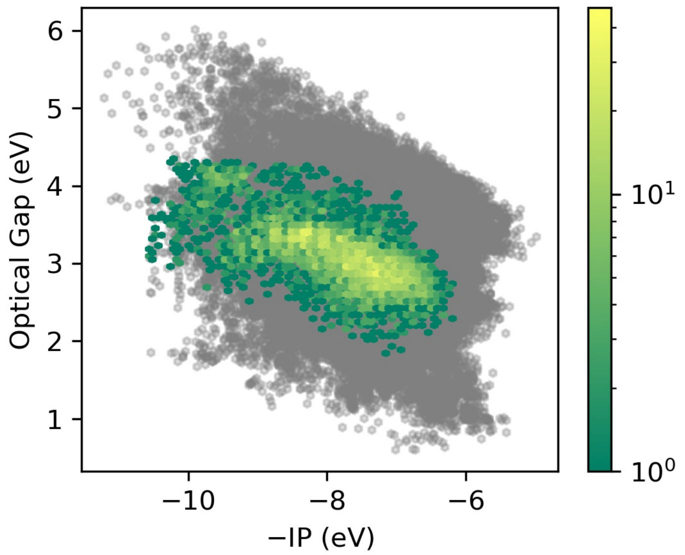

Supplementary Fig. 77 2D histogram of the property space spanned by -IP and the optical gap for molecules containing [n](:[n]):[o].

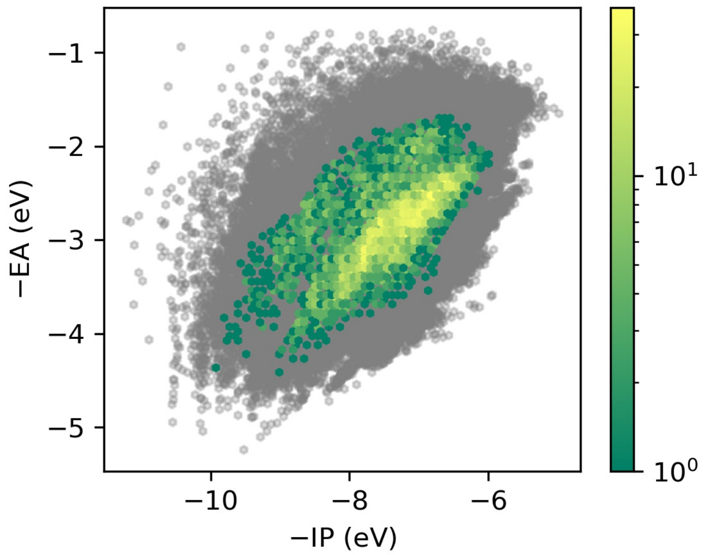

Supplementary Fig. 78 2D histogram of the property space spanned by -IP and -EA for molecules containing [n](:[cH]):[s].

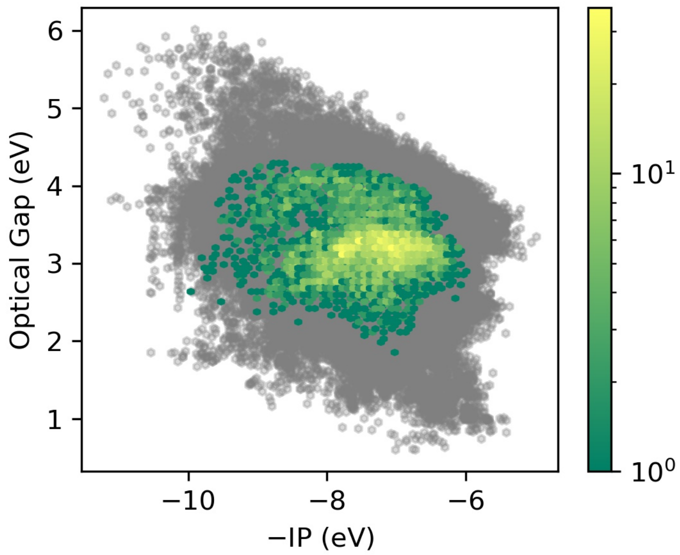

Supplementary Fig. 79 2D histogram of the property space spanned by -IP and the optical gap for molecules containing [n](:[cH]):[s].

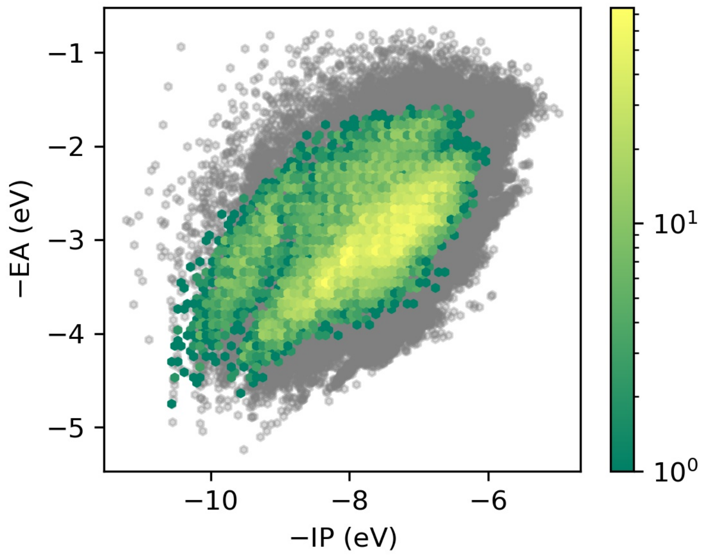

Supplementary Fig. 80 2D histogram of the property space spanned by -IP and -EA for molecules containing [n](:[cH]):[o].

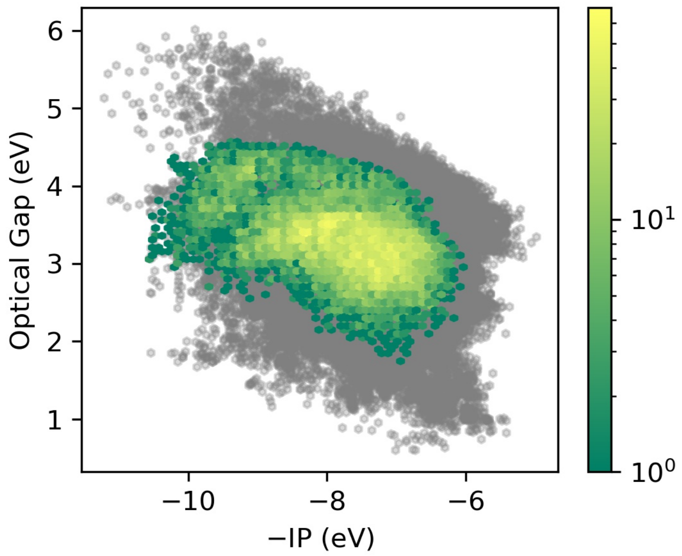

Supplementary Fig. 81 2D histogram of the property space spanned by -IP and the optical gap for molecules containing [n](:[cH]):[o].

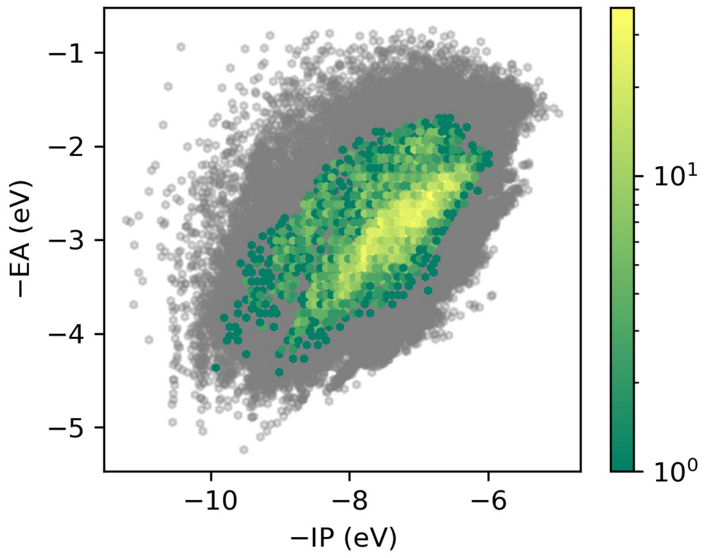

Supplementary Fig. 82 2D histogram of the property space spanned by -IP and -EA for molecules containing [cH](:[s]):[n].

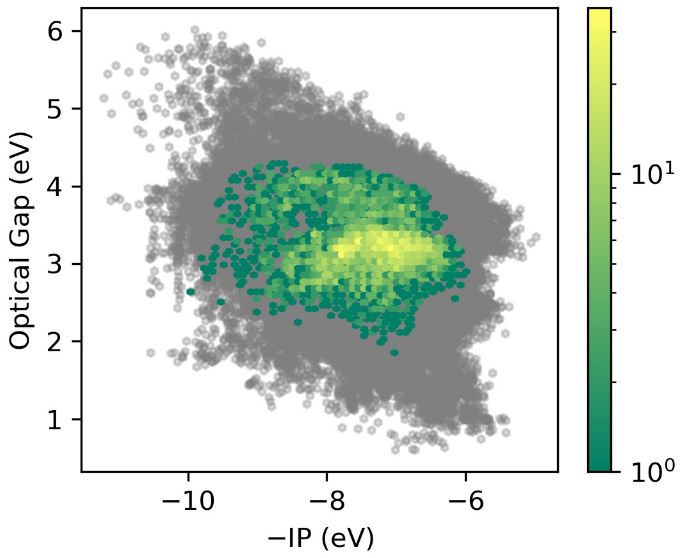

Supplementary Fig. 83 2D histogram of the property space spanned by -IP and the optical gap for molecules containing [cH](:[s]):[n].

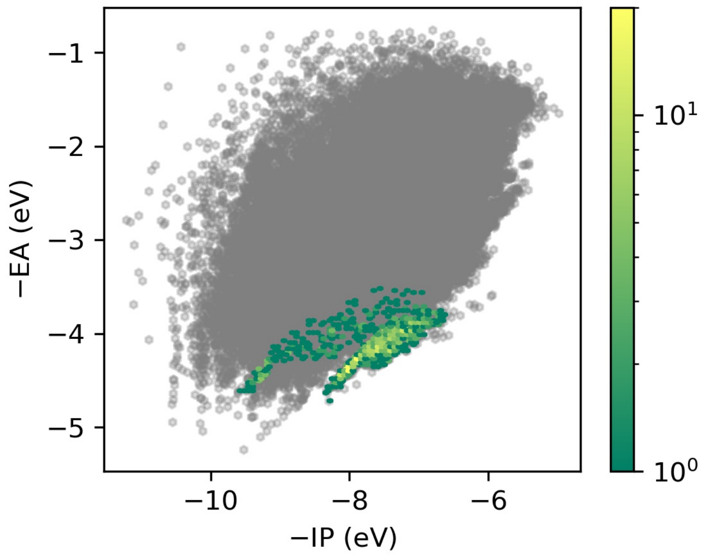

Supplementary Fig. 84 2D histogram of the property space spanned by -IP and -EA for molecules containing ([n](:[c])(:[c])-[CH3]).

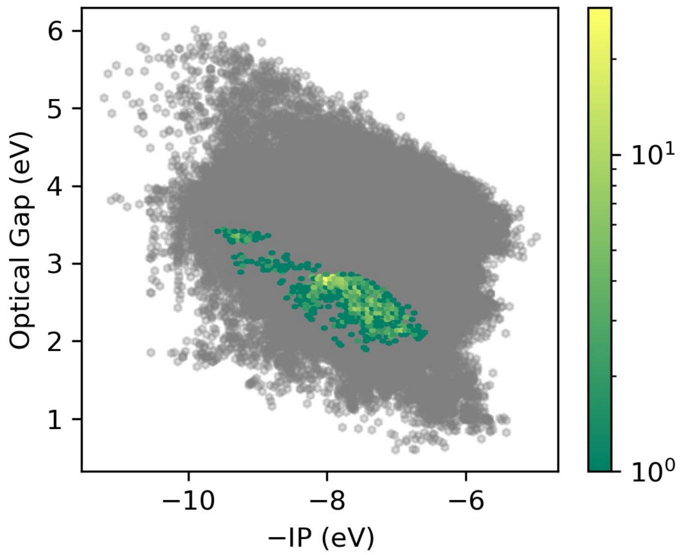

Supplementary Fig. 85 2D histogram of the property space spanned by -IP and the optical gap for molecules containing ([n](:[c])(:[c])-[CH3]).

Supplementary tables

Supplementary Table 1 Parameters of the linear model used to convert xTB values to the DFT scale, the corresponding coefficient of determination (r^2) and the mean average error (MAE).

	slope	intercept	r^2	MAE
-IP	1.076	0.151	0.888	0.20
-EA	0.821	0.616	0.957	0.12
optical gap	0.925	0.110	0.862	0.21

Supplementary Table 2 Prevalent skeletons identified through the topographical analysis and their corresponding -IP/-EA regions.

Most Prevalent Skeleton SMILES	-IP min	-IP max	-EA min	-EA max
c1nncc2nsnc12	-inf	-3.5	-inf	-3.5
c1nncc2oncc12	-inf	-3.5	-3.5	-2.5
c1nnco1	-inf	-3.5	-2.5	-1.5
c1nc2ccc3ncnc4ccc(n1)c2c34	-3.5	-2.5	-inf	-3.5
c1cnc2ncccc2c1	-3.5	-2.5	-3.5	-2.5
c1cncnc1	-3.5	-2.5	-2.5	-1.5
Cn1c(=O)c2ccc3c4ccc5c(=O)n(C)c(=O)c6ccc(c7ccc(c1=O)c2c37)c4c56	-2.5	-1.5	-inf	-3.5
c1nncc2cc3sncc3cc12	-2.5	-1.5	-3.5	-2.5
c1ccc2c(c1)Cc1ccccc1-2	-2.5	-1.5	-2.5	-1.5
c1nnc[nH]1	-2.5	-1.5	-1.5	-0.5
c1c2c(cc3nsnc13)N=S=N2	-1.5	-0.5	-inf	-3.5
c1ccc2cc3cc4ccccc4cc3cc2c1	-1.5	-0.5	-3.5	-2.5
c1cc2cc3sccc3cc2s1	-1.5	-0.5	-2.5	-1.5
c1cc[nH]c1	-1.5	-0.5	-1.5	-0.5
c1cc2[nH]c3cc[nH]c3c2[nH]1	-0.5	inf	-2.5	-1.5
c1cc2sc3cc[nH]c3c2[nH]1	-0.5	inf	-1.5	-0.5

Supplementary methods

SMILES fragment notation

In the main text we present these fragments as SMILES strings written in a condensed form, e.g. [cH][nH][cH], but with explicit hydrogen atoms, where the central atom of the fragment occurs in the middle of the string. The explicit hydrogen atoms are important as [cH][nH]c and c[nH]c, fragments where one or both carbon atoms besides the pyrrolic nitrogen have a substituent, are classed based on the radius 1 Morgan Extended-connectivity fingerprints as different fragments than [cH][nH][cH], as well as each other. Atoms that form part of an aromatic ring are shown in lowercase and aliphatic carbons in uppercase. However, the Morgan fingerprinting algorithm with radius 1 classifies the cC(=O)c fragment of anthraquinone and the CC(=O)C fragment of a benzoquinone molecule were both carbons adjacent to the central carbonyl group have been functionalised as the same fragment.
In the supporting figures we use in the captions of Figs. S28-S85 the long form of the SMILES with the central atom of the fragment on the right, e.g. [nH](:[nH]):[nH], where additionally single (-) and aromatic (:) bonds are explicitly shown.
