# Supplementary material for: Mapping the optoelectronic property space of small aromatic molecules
Source: Commun Chem. 2020 Feb 5;3:14. doi: 10.1038/s42004-020-0256-7 (PMC9814262; doi:10.1038/s42004-020-0256-7)
Supplement: Supplementary file 2 — Description of Additional Supplementary Files [file 42004_2020_256_MOESM2_ESM.docx]

**Description of Additional Supplementary Files**

File Name: Supplementary Data 1

Description: xTB and (TD-)DFT data for the subset of molecules used to calibrate xTB.

File Name: Supplementary Data 2

Description: Properties predicted by xTB, calibrated and uncalibrated, for all molecules in the data set, as well as their canonical SMILES.
